# A chromatin modifier integrates insulin/IGF‐1 signalling and dietary restriction to regulate longevity

**DOI:** 10.1111/acel.12477

**Published:** 2016-04-02

**Authors:** Anupama Singh, Neeraj Kumar, Latika Matai, Vaibhav Jain, Amit Garg, Arnab Mukhopadhyay

**Affiliations:** ^1^ Molecular Aging LaboratoryNational Institute of ImmunologyAruna Asaf Ali MargNew Delhi110067India; ^2^ CSIR‐Institute of Genomics & Integrative BiologySouth CampusMathura RoadNew Delhi110020India; ^3^ Academy of Scientific and Innovative ResearchCSIR‐IGIB, Mathura Road CampusNew DelhiIndia; ^4^ Present address: Centre for Human Genetics and Molecular MedicineSchool of Health SciencesCentral University of PunjabBathinda151001India

**Keywords:** *C. elegans*, dietary restriction, *daf‐16*, insulin signalling, incoherent feed‐forward loop, lifespan, *pha‐4*, *zfp‐1*

## Abstract

Insulin/IGF‐1‐like signalling (IIS) and dietary restriction (DR) are the two major modulatory pathways controlling longevity across species. Here, we show that both pathways license a common chromatin modifier, ZFP‐1/AF10. The downstream transcription factors of the IIS and select DR pathways, DAF‐16/FOXO or PHA‐4/FOXA, respectively, both transcriptionally regulate the expression of *zfp‐1*. ZFP‐1, in turn, negatively regulates the expression of DAF‐16/FOXO and PHA‐4/FOXA target genes, apparently forming feed‐forward loops that control the amplitude as well as the duration of gene expression. We show that ZFP‐1 mediates this regulation by negatively influencing the recruitment of DAF‐16/FOXO and PHA‐4/FOXA to their target promoters. Consequently, *zfp‐1* is required for the enhanced longevity observed during DR and on knockdown of IIS. Our data reveal how two distinct sensor pathways control an overlapping set of genes, using different downstream transcription factors, integrating potentially diverse and temporally distinct nutritional situations.

## Introduction

Organisms may possess an intrinsic ability to modulate lifespan in response to the nutritional status of the environmental niche they reside in. This is reflected in the fact that modulations of the conserved insulin/IGF‐1 signalling (IIS) pathway or dietary/caloric restriction (DR) lead to dramatic increases in longevity across species (Kenyon, 2005, 2010). Perfunctorily, it would appear that the modulatory signalling through the IIS pathway that couples nutrient status to aging will generally overlap with the mechanisms by which DR affects lifespan. However, earlier research in *Caenorhabditis elegans* as well as in *Drosophila melanogaster* has shown that their relationship is rather complicated. Lifespan extension in the case of the IIS signalling mutants is strictly dependent on the FOXO transcription factor DAF‐16, while different DR regimes have varied requirements for downstream transcription factors (Greer *et al*., 2007a; Panowski *et al*., 2007; Greer & Brunet, 2009; Kenyon, 2010). For example, DAF‐16 is required for lifespan extension when DR is initiated by bacterial dilution on solid plates (sDR) or by the dilution of peptone (Greer & Brunet, 2009). On the other hand, in *C. elegans*, a FOXA transcription factor PHA‐4 is required for two other types of DR‐induced longevity, namely the bacterial dilution in liquid culture (bDR) and in the *eat‐2* mutant (Panowski *et al*., 2007). However, PHA‐4 is not required for IIS pathway mutants to increase lifespan (Panowski *et al*., 2007). Further, genetically combining an IIS mutant with an *eat‐2* mutant increased lifespan over and above that of the individual long‐lived mutants, suggesting independent mechanisms (Lakowski & Hekimi, 1998). Additionally, these pathways seem to have developed extensive mechanisms of crosstalks involving other transcriptional regulators. A FOXO/DAF‐16 coregulator SMK‐1 is required for the DR‐mediated lifespan extension in *C. elegans*, although the mechanism has not been elucidated (Wolff *et al*., 2006). Also, the TFEB orthologue HLH‐30 and NRF2‐like transcription factor SKN‐1 function downstream of both the pathways (Bishop & Guarente, 2007; Tullet *et al*., 2008; Lapierre *et al*., 2013) to regulate lifespan through distinct mechanisms. Thus, there may be other possible common players integrating IIS and DR signalling that needs to be deciphered in order to understand their complex relationship.

Here, we identify a chromatin‐associated factor ZFP‐1/AF10 as a common mediator of IIS‐ and DR‐mediated lifespan regulation. ZFP‐1 is the homolog of AF10, a zinc finger protein that has roles in RNA interference (RNAi) and development (Grishok *et al*., 2008; Avgousti *et al*., 2013). In mammals, AF10 is best known as a fusion partner of the mixed lineage leukaemia (*MLL*) or clathrin‐associated lymphoid myeloids (*CALM*) genes that lead to acute leukaemia, mostly in infants. Fused MLL‐AF10 or CALM‐AF10 results in dysregulation of the *HOX* gene cluster in conjunction with *MEIS1* and may be the putative mechanism of leukaemia (Caudell & Aplan, 2008). AF10/ZFP‐1 is known to interact with H3K79 methyltransferase Dot‐1‐like (DOT1L; DOT‐1 in *C. elegans*) using its octapeptide motif‐leucine zipper domain (OM‐LZ) as well as with GLIOMA‐AMPLIFIED SEQUENCE‐41 (GAS41; GFL‐1 in *C. elegans*) (Debernardi *et al*., 2002; Cecere *et al*., 2013). The ZFP‐1(AF10)/DOT‐1 complex is involved in RNA polymerase II pausing during development and stress by modifying H3K79 and consequently H2B monoubiquitination patterns (Cecere *et al*., 2013). ZFP‐1 has also been found to utilize its PHD1–PHD2 domains to interact with lysine 4‐methylated histones and is required during embryogenesis (Avgousti *et al*., 2013). GAS41 is similar to AF9, another fusion partner of MLL and interacts with the SWI/SNF complex (Debernardi *et al*., 2002). Interestingly, DAF‐16 has been shown recently to promote stress resistance and longevity by employing the SWI/SNF complex (Riedel *et al*., 2013).

In an earlier study, ZFP‐1 emerged as a strong direct target of DAF‐16/FOXO (Oh *et al*., 2006). In this study, we demonstrate that both DAF‐16/FOXO and PHA‐4/FOXA directly bind and regulate the expression of the different isoforms of *zfp‐1* as well as *gfl‐1*. Interestingly, ZFP‐1/GFL‐1 in turn negatively regulates the expression of DAF‐16/FOXO and PHA‐4/FOXA target genes, forming apparent incoherent feed‐forward loops (FFLs). We provide evidence that ZFP‐1/GFL‐1 determines the amplitude and duration of target gene expression during low IIS or DR. We show that ZFP‐1 negatively influences the recruitment of DAF‐16 and PHA‐4 to the promoters of their direct target genes, thereby repressing their transcription. Knocking down *zfp‐1* or *gfl‐1* may therefore lead to large‐scale deregulation of DAF‐16 or PHA‐4 target gene expression under low IIS or DR, respectively. Consequently, ZFP‐1 and GFL‐1 are required for IIS‐ and DR‐mediated longevity assurance. Our study elucidates how two sensor pathways, processing possibly diverse nutrient information cues, converge on a single chromatin‐associated factor to fine‐tune the expression of an overlapping set of genes. Because DAF‐16, PHA‐4 and ZFP‐1 are highly conserved proteins, it is possible that such ZFP‐1/GFL‐1‐mediated fine‐tuning of downstream target gene expression is commonly used by IIS and DR in higher mammals.

## Results

### DAF‐16/FOXO regulates different isoforms of *zfp‐1* as well as *gfl‐1*

**Figure 1:**
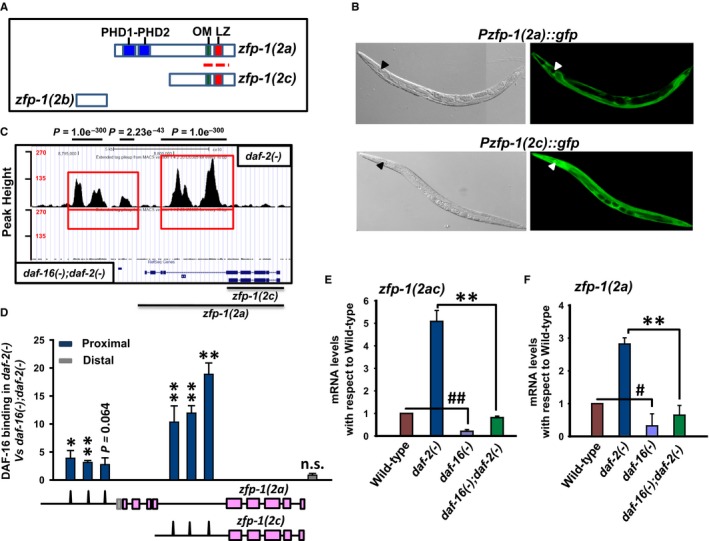
The isoforms of worm AF10 ortholog are direct transcriptional target of DAF‐16/FOXO. (A) Organization of ZFP‐1 isoforms. The polypeptide encoded by *zfp‐1(2a)* isoform possesses the PHD domain, OM (octapeptide motif) and LZ (leucine zipper) motifs, whereas the shorter isoform encoded by *zfp‐1(2c)* has the OM‐LZ motif only. The third isoform *zfp‐1(2b)* lacks all these domains and motifs. Red dotted line indicates region from where RNAi was designed. (B) Expression pattern of GFP driven by *zfp‐1(2a)* or *zfp‐1(2c)* promoters (right panels). Corresponding DIC images are shown in the left panels. Pharynx is marked by arrow head. (C) UCSC browser view of DAF‐16/FOXO peaks as determined by ChIP‐seq using anti‐DAF‐16/FOXO antibody (Kumar *et al*., 2015). Red boxes indicate the promoter regions of *zfp‐1(2a)* and *zfp‐1(2c)* where DAF‐16/FOXO peaks are observed. Lower panel shows *daf‐16(mgDf50);daf‐2(e1370)* [represented as *daf‐16(‐);daf‐2(‐)*] that lacks specific DAF‐16/FOXO peaks, while upper panel shows peaks obtained in *daf‐2(e1370)* [represented as *daf‐2(‐)*] (D) ChIP‐PCR validation of DAF‐16/FOXO binding to *zfp‐1* promoters obtained by ChIP‐seq. Binding in *daf‐2(‐)* is normalized to that of *daf‐16(‐);daf‐2(‐)*. The corresponding peaks on the promoters of *zfp‐1*, as obtained by ChIP‐seq, are shown pictographically below the graph. (E‐F) quantitative RT (QRT)–PCR detection of transcript levels for *zfp‐1(2ac)* or *zfp‐1(2a)* in WT and different mutants as mentioned. The *zfp‐1(2c)* transcript cannot be detected separately from *zfp‐1(2a)* as explained in the text. Error bars are standard deviation. **P ≤* *0.01; *P ≤* *0.05; n.s., *P* not significant by Student's *t‐*test. ^##^ *P ≤ *0.01; ^#^ *P ≤ *0.05 compared to wild‐type. The graphs were plotted from three experiments.

The *zfp‐1* gene encodes three distinct isoforms (Figure 1A). The ZFP‐1(2a) protein has two conserved domains, the PHD1–PHD2 zinc finger and the OM‐LZ motif, while ZFP‐1(2c) lacks the PHD1–PHD2 domain (Mansisidor *et al*., 2011; Avgousti *et al*., 2013). A third isoform, ZFP‐1(2b) also exists but lacks both the domains and was not considered in this study. Each isoform has distinct SL1 sites (Figure S1A) and their individual promoters can drive the expression of GFP in a tissue‐specific manner (Fig. 1B). While the *zfp‐1(2c)* promoter drove GFP expression uniformly in the worm, the *zfp‐1(2a)* promoter‐driven expression was noticeably absent from the pharynx, germline and the tail regions. A genome‐wide endogenous DAF‐16/FOXO ChIP sequencing study in our laboratory (Kumar *et al*., 2015) showed that the transcription factor binds to the promoters of both the *zfp‐1* isoforms in the temperature‐sensitive *daf‐2(e1370)* allele of *daf‐2* (Fig. 1C; see supplementary Materials and methods for analysis details and data access links). Three peaks each were observed on the promoters of both *zfp‐1(2a)* and *zfp‐1(2c)* (Fig. 1C). The *daf‐2* gene codes for the IIS receptor in worms that negatively regulates DAF‐16/FOXO through a conserved signalling cascade (Kenyon, 2010). The *daf‐2(e1370)* allele, where DAF‐16 is in an activated state, has extended lifespan, enhanced stress tolerance and at 25 °C arrests as dauers, an alternative developmental stage controlled by the IIS, while at 15 or 20 °C, it enters reproductive development. We validated the binding of DAF‐16 to the individual promoter regions by ChIP‐PCR (Fig. 1D). We further investigated whether each of these isoforms are transcriptionally dependent on DAF‐16. It is not possible to separately detect the *zfp‐1(2c)* isoform as it has 100% overlap with *zfp‐1(2a)*; the *2a* isoform has additional 5′ exons [Fig. 1A,D (lower panel)]. We therefore designed primers specific to the *zfp‐1(2a)* isoform and a pair that detected the *zfp‐1(2a)* together with the *zfp‐1(2c)* isoform (referred to as *2ac*). The expression of *zfp‐1(2c)* may then be deduced by subtracting the *zfp‐1(2a)* expression values from that of *zfp‐1(2ac)*. We compared the expression of these isoforms between wild‐type, *daf‐2(e1370)* [represented from now on as *daf‐2(‐)* for convenience although it is not a null mutant at 20 °C], *daf‐16(mgDf50)* [*daf‐16(‐)*] and *daf‐16(mgDf50);daf‐2(e1370)* [*daf‐16(‐); daf‐2(‐)*], grown at 20 °C using quantitative RT–PCR (QRT–PCR). We found that both the isoforms are upregulated in *daf‐2(‐)* compared to wild‐type, in a *daf‐16*‐dependent manner (Fig. 1E,F). The basal expression of *zfp‐1(2a)* and *zfp‐1(2ac)* genes is also dependent on *daf‐16*. Together, the isoforms of *zfp‐1* are direct transcriptional targets of DAF‐16.

In mammals, AF10 physically associates with GAS41, a protein that interacts with the human SWI/SNF complex (Debernardi *et al*., 2002). DAF‐16 ChIP‐seq data indicated that *gfl‐1* (the *C. elegans* gene that codes for the GAS41 homolog) promoter is bound by DAF‐16 (Figure S1B). We verified the binding by ChIP‐PCR using primers designed against the single DAF‐16 binding site (Figure S1C). Additionally, the expression of *gfl‐1* in *daf‐2(‐)* is dependent on *daf‐16*, although in wild‐type it is not (Figure S1D). Together, *zfp‐1* and its interactor, *gfl‐1* (that has overlapping expression pattern with *zfp‐1* isoforms; Figure S1E), are direct targets of DAF‐16, downstream of the IIS pathway.

### Differential regulation of *zfp‐1* isoforms by different DAF‐16/FOXO isoforms

ZFP‐1 is a strong target of DAF‐16/FOXO (Oh *et al*., 2006), indicating that it may carry out important functions downstream of the transcription factor. The *daf‐16* gene codes for several distinct and well‐characterized isoforms (Lee *et al*., 2001; Lin *et al*., 2001; Kwon *et al*., 2010). The *daf‐16(a)* isoform is coded by R13H8.1b, the *daf‐16(b)* by R13H8.1a and the *daf‐16(f)* by R13H8.1f. We next asked whether all the DAF‐16 isoforms regulate the ZFP‐1 isoforms, attesting to the importance of this downstream target. We used integrated strains where *daf‐16(a)* (HT1881), *daf‐16(b)* (HT1882) or *daf‐16(f)* (HT1883) is rescued in *daf‐16(‐);daf‐2(‐)* (Kwon *et al*., 2010) and determined binding of these isoforms to *zfp‐1* or *gfl‐1* promoters (Figures S2A and S3A). We found that all the isoforms of DAF‐16 bind to the promoters of *zfp‐1* or *gfl‐1* to varying extent (Figures S2A, S3A). QRT–PCR analysis revealed that all the *zfp‐1* isoforms as well as *gfl‐1* are transcriptionally regulated by all the three DAF‐16 isoforms (Figures S2B and S3B). These observations supported our assumption that ZFP‐1 and GFL‐1 may be important DAF‐16/FOXO targets.

### The FOXA transcription factor PHA‐4 also regulates *zfp‐1* and *gfl‐1* gene expression

**Figure 2:**
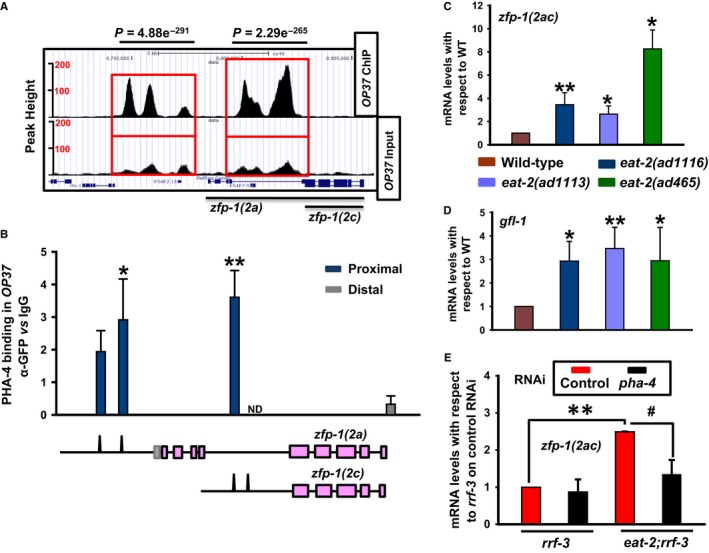
Dietary restriction (DR)‐specific transcription factor PHA‐4/FOXA also directly regulates *zfp‐1* and *gfl‐1*. (A) UCSC browser view of PHA‐4/FOXA peaks on *zfp‐1(2a)* and *zfp‐1(2c)* promoters as determined by ChIP‐seq analysis of *unc‐119(ed3) III; wgIs37* (*OP37*) strain; data mined from MODENCODE and reanalysed using our bioinformatic pipeline. Red boxes indicate the promoter regions of *zfp‐1(2a)* and *zfp‐1(2c)* where peaks are observed. Lower panel shows peaks in input samples. (B) ChIP‐PCR validation of PHA‐4/FOXA binding to *zfp‐1* promoters obtained by ChIP‐seq analysis of *OP37* strain. Enrichment using GFP antibody when normalized to that of IgG is plotted in the *Y*‐axis. The corresponding peaks on the promoters of *zfp‐1*, as obtained by ChIP‐seq (MODENCODE), are shown pictographically below the graph. ND – not determined. (C,D) Quantitative RT (QRT)–PCR analysis of the transcript levels of *zfp‐1(2ac)* (C) or *gfl‐1* (D) in different *eat‐2* mutant strains. The graph is plotted from three or more experiments. (E) QRT–PCR detection of transcript levels for *zfp‐1(2ac)* in *rrf‐3(pk1426)* or *eat‐2(ad1116);rrf‐3(pk1426)* grown on control or *pha‐4* RNAi. Error bars are standard deviation. **P ≤ 0.01;*P ≤* *0.05; ^#^ *P ≤ *0.05 by Student's *t‐*test.

Forkhead transcription factors play important roles in regulating longevity in *C. elegans*. While DAF‐16/FOXO acts mostly downstream of the IIS (Kenyon, 2010) and in some forms of DR (Greer & Brunet, 2009), PHA‐4/FOXA is required for two other paradigms of DR‐mediated longevity, but not for IIS (Panowski *et al*., 2007). As *zfp‐1* and *gfl‐1* were found to be robust targets of DAF‐16/FOXO, we asked whether they are also regulated by the FOXA transcription factor. In worms, DR regimes that require PHA‐4 can be initiated either by using the *eat‐2* mutants or by diluting the bacterial feed (Panowski *et al*., 2007). The *eat‐2* mutants have defective pharyngeal pumping that lead to lower food intake and are considered to mimic DR (Lakowski & Hekimi, 1998). We reanalysed the PHA‐4 ChIP‐seq data available at MODENCODE (Zhong *et al*., 2010) using our analysis pipeline and found that *zfp‐1* and *gfl‐1* promoters are directly bound by PHA‐4 (Figs 2A and S4A). While two distinct PHA‐4 binding peaks were observed on the *zfp‐1(2a)* and *zfp‐1(2c)* promoters, *gfl‐1* promoter possessed a single peak (Figs 2A and S4A). We validated the recruitment of PHA‐4 to the individual promoter regions by ChIP QRT–PCR in *unc‐119(ed3) III; wgIs37* (OP37) strain using anti‐GFP antibody (Fig. 2B). Consequently, the expression of the *zfp‐1* isoforms and *gfl‐1* is elevated in *eat‐2(ad1116)* as well as in *eat‐2(ad1113)* and *eat‐2(ad465)*, compared to wild‐type (Figs 2C,D and S4B). This increased expression was dependent on PHA‐4 (Figs 2E, S4C and S4D). Importantly, in another newly identified DR‐like paradigm (Chamoli *et al*., 2014), knocking down a *mekk‐3*‐like gene *drl‐1* led to an increased expression of the *zfp‐1* isoforms, but not *gfl‐1* (Figure S4E). Together, these data show that similar to DAF‐16/FOXO, PHA‐4/FOXA also transcriptionally regulates the expression of *zfp‐1* and *gfl‐1*.

### ZFP‐1 regulates DAF‐16 target gene expression

**Figure 3:**
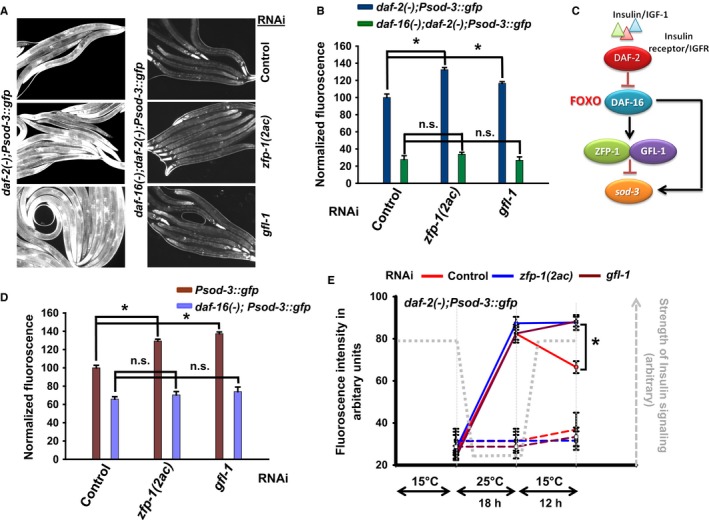
ZFP‐1 and GFL‐1 negatively regulate *sod‐3*, a positively regulated DAF‐16/FOXO direct target, forming a type 1 incoherent feed‐forward loop. (A) *daf‐2(‐)*;*Psod‐3::gfp* or *daf‐16(‐);daf‐2(‐);Psod‐3::gfp* was grown on control, *zfp‐1(2ac)* or *gfl‐1* RNAi and images captured under fluorescence microscope at 100× magnification. (B) Quantification of GFP fluorescence as observed in (A) using ImageJ. Fluorescence was normalized to *daf‐2(‐)*;*Psod‐3::gfp* on control RNAi. The error bars indicate SEM between two or more experiments. (C) A circuit diagram showing a type I incoherent feed‐forward loop involving ZFP‐1, DAF‐16/FOXO and *sod‐3*. (D) Quantification of GFP fluorescence in *Psod‐3::gfp* or *daf‐16(‐);Psod‐3::gfp* on control, *zfp‐1(2ac)* or *gfl‐1* RNAi. Fluorescence was normalized to *Psod‐3::gfp* on control RNAi. The error bars indicate SEM between three experiments. (E) *daf‐2(‐)*;*Psod‐3::gfp* worms were grown at 15 °C on control, *zfp‐1(2ac)* or *gfl‐1* RNAi since hatching. L3 worms were shifted to 25 °C for 18 h and then shifted back to 15 °C. At the mentioned time points, images were acquired using a fluorescence microscope and quantified using ImageJ. Data shown are from one of two experiments. Dotted lines represent the worms maintained at 15 °C throughout on respective RNAi. The error bars indicate standard error mean for *n* > 25. *P ≤* *0.05 by Student's *t‐*test, n.s., non‐significant.

Why would DAF‐16/FOXO regulate the transcription of chromatin modifiers like ZFP‐1/GFL‐1? We hypothesized that because DAF‐16 and ZFP‐1 may be part of a larger complex (Riedel *et al*., 2013), they would possibly influence the expression of DAF‐16 target genes. To study this aspect of regulation, we focused on the well‐known DAF‐16 direct target, *sod‐3* (Oh *et al*., 2006; Mukhopadhyay *et al*., 2008; Kumar *et al*., 2015). Under conditions of low insulin signalling, as seen in *daf‐2(‐)*,* sod‐3* is chronically upregulated in a *daf‐16*‐dependent manner (Libina *et al*., 2003). The dynamics of this regulation can be studied by using an integrated *Psod‐3::gfp* transgenic strain (Libina *et al*., 2003). We grew *Psod‐3::gfp, daf‐16(‐);Psod‐3::gfp, daf‐2(‐);Psod‐3::gfp* or *daf‐16(‐);daf‐2(‐);Psod‐3::gfp* on control, *zfp‐1(2ac)* or *gfl‐1* RNAi and quantified the GFP fluorescence of the worms. The *zfp‐1(2ac)* RNAi effectively knocks down the expression of both the *2a* and the *2c* transcripts, while the *gfl‐1* RNAi significantly reduced *gfl‐1* mRNA levels (Figure S4F, G). We found that knocking down the *zfp‐1(2ac)* as well as *gfl‐1* increased the expression of *Psod‐3::gfp* in WT as well as in *daf‐2(‐)* background and this was dependent on *daf‐16* as we observed no increase in *daf‐16(‐);Psod‐3::gfp* or *daf‐16(‐);daf‐2(‐);Psod‐3::gfp* (Fig. 3A,B,D). QRT–PCR analysis of *sod‐3* mRNA expression in WT and *daf‐2(‐)* also showed a similar upregulation when *zfp‐1* was knocked down (Figure S5A). Thus, DAF‐16 positively controls the expression of *sod‐3* as well as *zfp‐1*. However, ZFP‐1/GFL‐1 negatively regulates the expression of *sod‐3*. Therefore, these genes form a typical type I incoherent feed‐forward loop (FFL) (Mangan & Alon, 2003; Alon, 2007) downstream of the IIS (Fig. 3C). This type I incoherent FFL has two transcriptional regulators, one (DAF‐16) positively regulating the other (ZFP‐1). These factors then regulate a common transcriptional target *sod‐3*; DAF‐16 controls it positively, while ZFP‐1 negatively modulates it.

To determine whether such a mode of regulation exists only in the case of *sod‐3*, we checked several other genes regulated by DAF‐16 (Kwon *et al*., 2010; Zhang *et al*., 2013) and observed a similar upregulation of expression in the case of *mtl‐1, lys‐7*,* ZK742.4, hsp‐12.6*,* sod‐5, scl‐1, sip‐1, dod‐11, gpd‐2* as well as *hsp‐16.2* when *zfp‐1(2ac)* was knocked down (Figure S5A–C). This showed that DAF‐16 and ZFP‐1 may regulate many other genes using FFLs. However, we found that *gfl‐1* knockdown did not significantly change expression in the case of *mtl‐1, hsp‐16.2* and *gpd‐2* (Figure S5B), but had a more robust response in the case of *dod‐11* (Figure S5C), suggesting that GFL‐1 and ZFP‐1 may collaborate in some contexts, but not in others.

### ZFP‐1 determines the duration of expression of DAF‐16 target genes

Incoherent FFLs typically function to control the amplitude and duration of gene expression (Alon, 2007). We asked whether the DAF‐16:ZFP‐1:SOD‐3 FFL performs similar function downstream of the IIS pathway. By shifting the temperature‐sensitive *daf‐2(‐)* between 15 and 25 °C, we could modulate the IIS and as a consequence, the *sod‐3::gfp* expression in the mutant. The *daf‐2(‐);Psod‐3::gfp* worms were grown at 15 °C on control RNAi, *zfp‐1(2ac)* or *gfl‐1* RNAi from hatching and then shifted to 25 °C for 18 h at L2 stage. This step led to an enhanced *sod‐3::gfp* expression, possibly due to further lowering of the IIS and/or activation of DAF‐16 (Fig. 3E). Worms that were continuously maintained at 15 °C did not exhibit the surge in GFP expression (Fig. 3E). These worms were then returned to 15 °C, and the expression of *Psod‐3::gfp* was monitored after 12 h. We found that while the expression of *Psod‐3::gfp* declined when the worms were restored to 15 °C on control RNAi, expression in the ones grown on *zfp‐1(2ac)* or *gfl‐1* RNAi failed to decrease to similar extent (Fig. 3E). We performed similar experiments using QRT–PCR to detect mRNA levels of *sod‐3* and *mtl‐1*; the fall in the mRNA expression levels was slower in the case of *zfp‐1* as well as *gfl‐1* knockdown (Figure S5D). Thus, ZFP‐1 and GFL‐1 regulate the duration of *sod‐3* gene expression, apart from amplitude, following the activation of DAF‐16/FOXO.

### ZFP‐1 regulates the extent of DAF‐16 binding to target promoters

**Figure 4:**
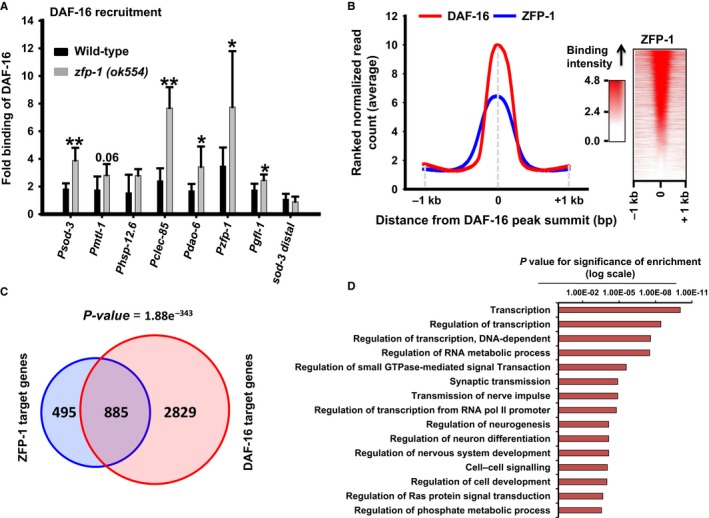
ZFP‐1 influences DAF‐16/FOXO recruitment to its target promoters. (A) ChIP‐PCR to determine DAF‐16 recruitment to different target promoters in WT and *zfp‐1(ok554)*. Binding in WT */zfp‐1(ok554)* is normalized to that of *daf‐16(‐)*. Recruitment at a distal region of *sod‐3* is taken as control. The graph is plotted from three experiments. Error bars represent standard deviation. **P ≤ 0.01; *P ≤* *0.05 by Student's *t‐*test. (B) Distribution of ZFP‐1 peaks with respect to DAF‐16/FOXO binding summits as determined by ChIP‐seq experiments (left panel). Distribution of ZFP‐1 reads with respect to DAF‐16 binding summit across all chromosomes (right panel). ZFP‐1 ChIP‐seq data from MODENCODE were reanalysed using our bioinformatic pipeline. (C) Overlap of ZFP‐1 and DAF‐16 direct target genes as determined by ChIP‐seq experiments. (D) Gene Ontology (GO) term analysis of common target genes of DAF‐16/FOXO and ZFP‐1. Only top 15 GO terms that were enriched are shown.

We found that knocking down the *zfp‐1(2ac)* isoform leads to an increased expression of DAF‐16 direct targets like *sod‐3* and *mtl‐1*. In order to understand the nature of this regulation, we asked whether the recruitment of DAF‐16 is influenced by ZFP‐1. For this, we performed ChIP using anti‐DAF‐16 antibody in wild‐type, *zfp‐1(ok554)* as well as *daf‐16(‐),* and evaluated DAF‐16 binding at the *sod‐3* promoter by quantitative PCR. We found that binding of DAF‐16 increased significantly in the absence of ZFP‐1, on the *sod‐3* promoter (Fig. 4A). However, no increase was seen in the distal region of *sod‐3*. This correlates well with the increase in *sod‐3* expression on *zfp‐1(2ac)* knockdown and showed that ZFP‐1 negatively regulates the direct binding of DAF‐16 to the *sod‐3* promoter. Similar observations were made in the case of other DAF‐16 targets (Fig. 4A). Interestingly, ZFP‐1 also negatively regulates the binding of DAF‐16 to its own promoter, forming a feed‐back loop as shown earlier (Cecere *et al*., 2013). We were unable to generate a *daf‐2(e1370);zfp‐1(ok554)* or *daf‐2(e1368);zfp‐1(ok554)* double mutant due to larval lethality, as reported previously (Mansisidor *et al*., 2011), but anticipate similar mechanism in a *daf‐2(‐)* scenario. Thus, ZFP‐1 may fine‐tune DAF‐16‐dependent expression of genes by regulating the binding of the transcription factor to its target promoters.

Next, we asked whether this phenomenon is restricted to *sod‐3* and a few other DAF‐16 direct targets or is a more global feature. For this, we reanalysed the ChIP‐seq data for ZFP‐1 available at MODENCODE (seq‐JL00006_ZFP1_N2_L3) and compared it with that of our DAF‐16 ChIP‐seq data (Kumar *et al*., 2015). We found that the ZFP‐1 peaks on the chromatin colocalized with the DAF‐16 peak summits over the entire genome (Fig. 4B, left panel). This indicated that DAF‐16 and ZFP‐1 binds to similar regions on the chromatin. However, not all DAF‐16 peaks overlap with those of ZFP‐1 (Fig. 4B, right panel), suggesting that other DAF‐16 direct targets may be regulated differently. Alternatively, it is possible that because the DAF‐16 and ZFP‐1 ChIP‐seq experiments were performed in different genetic backgrounds and developmental stages, not all peaks show an overlap. Additionally, it appears that the ZFP‐1 footprint on DAF‐16 target promoters may vary depending on the target genes as we observed narrow as well as broad spread of ZFP‐1 binding with respect to DAF‐16 summits (Fig. 4B, right panel). Together, ZFP‐1 may bind to a large number of regions where DAF‐16/FOXO binds in the genome and influences the recruitment of the latter to the chromatin.

### Genes targeted by both DAF‐16 and ZFP‐1

Because ZFP‐1, a direct target of DAF‐16/FOXO, also influences the binding of DAF‐16 to its target promoters and forms feed‐forward loops, we asked what genes are cotargeted by the two proteins. For this, we only considered the genes where DAF‐16 and ZFP‐1 peaks are situated within the 2.0 kb of the promoter proximal region. We found that ZFP‐1 binds to the promoters of 1380 genes, while DAF‐16 peaks were found on 3714 gene promoters (Fig. 4C). However, a total of 885 genes were targeted by both DAF‐16 and ZFP‐1 (*P *=* *1.88e‐343 by hypergeometric test) that may constitute the feed‐forward loops. We used the Database for Annotation, Visualization and Integrated Discovery (DAVID) v6.7 package (Dennis *et al*., 2003) to analyse Gene Ontology (GO) term enrichment in these 885 common genes to predict their biological functions. We found that these genes are mostly enriched for molecular components required for transcriptional regulation, signal transduction and neurogenesis/neural signalling (Fig. 4D, Table S4). In fact, transcriptional regulators like nuclear hormone receptor (*P = *9.42E‐11), zinc finger proteins (*P = *4.55E‐11) and signalling proteins like serine/threonine protein kinases (*P = *5.32E‐07), Pleckstrin homology (PH) domain possessing proteins (*P = *6.81E‐05), Src homology‐3 (SH3) domain containing proteins (*P = *2.96E‐04) as well as EF‐hand proteins (*P = *3.57E‐04) are significantly enriched. Pathway analysis using Kyoto Encyclopaedia of Genes and Genomes (KEGG) database revealed an enrichment of components of MAPK signalling (*P = *1.60E‐04), mTOR signalling (*P = *1.10E‐03) as well as Jak‐STAT signalling (*P = *3.60E‐03) pathways. These data indicate that DAF‐16 and ZFP‐1 coregulate genes that may be important for longevity effects seen in *daf‐2(‐)*. In fact, 62 genes among the common DAF‐16 and ZFP‐1 targets have known function in aging (*P *=* *4.34 e‐7) according to the GenAge database (http://genomics.senescence.info/genes/), while genes involved in aging and lifespan regulation were enriched in the GO term analysis (*P *=* *3.90E‐02) (Table S4).

### PHA‐4/FOXA and ZFP‐1 also constitute FFLs downstream of DR signalling

**Figure 5:**
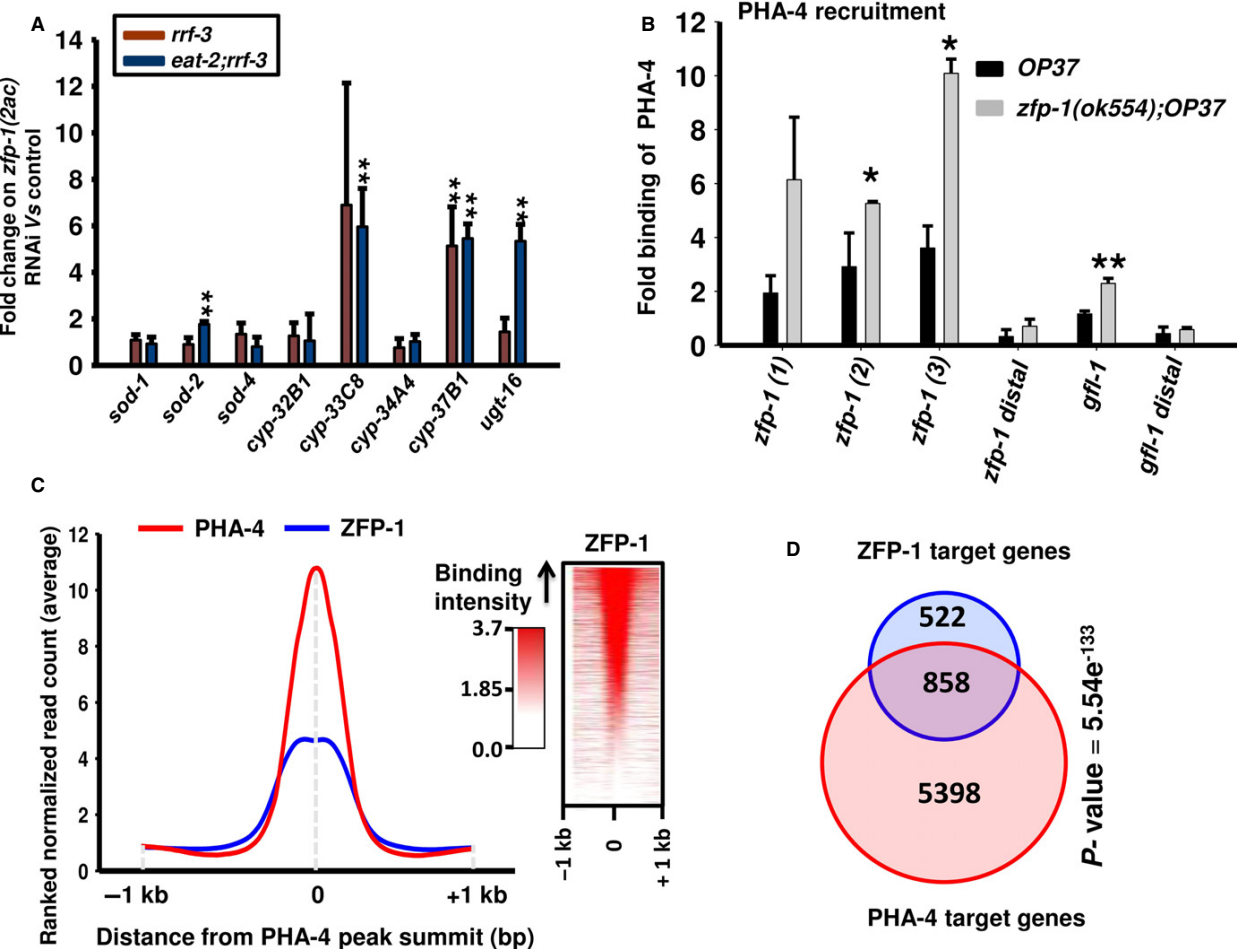
ZFP‐1 regulates expression of PHA‐4/FOXA target genes, forming incoherent feed‐forward loops (FFLs). (A) Relative expression levels of PHA‐4 target genes in *rrf‐3(pk1426)* or *eat‐2(ad1116);rrf‐3(pk1426)* grown on control vs *zfp‐1(2ac)* RNAi. (B) ChIP‐PCR to determine PHA‐4 recruitment to different target promoters in *unc‐119(ed3) III; wgIs37 (OP37)* and *zfp‐1(ok554);OP37*. Enrichment using anti‐GFP antibody when normalized to that of IgG control is plotted on the *Y*‐axis. Recruitment at *zfp‐1* (three peak regions) or *gfl‐1* promoters was quantified, while distal regions of *zfp‐1* and *gfl‐1* were taken as control. The graph is plotted from three experiments. Error bars are standard deviation. **P ≤* *0.01; *P ≤* *0.05 by Student's *t‐*test. (C) Distribution of ZFP‐1 peaks with respect to PHA‐4/FOXA binding summits as determined by reanalysis of previously published ChIP‐seq data available at MODENCODE (left panel). Distribution of ZFP‐1 reads with respect to PHA‐4/FOXA binding summit across all chromosomes (right panel). (D) Overlap of ZFP‐1 and PHA‐4/FOXA direct target genes as determined by the ChIP‐seq experiments.

Next, we determined whether ZFP‐1 is also a part of a regulatory loop involving PHA‐4/FOXA, similar to the case of DAF‐16/FOXO. For this, we chose a few published targets of PHA‐4 like *sod‐1, sod‐2, sod‐4* (Panowski *et al*., 2007), *cyp‐32B1, cyp‐33C8, cyp‐34A4*,* cyp‐37B1* and *ugt‐16* (Chamoli *et al*., 2014). Knocking down *zfp‐1(2ac)* significantly increased the expression of several of these genes in the *eat‐2* mutant, thereby showing that these genes may also form FFLs downstream of DR (Fig. 5A). We found that knocking down *zfp‐1* also leads to a greater enrichment of PHA‐4 at its target promoters, as in the case of DAF‐16 (Fig. 5B). Although we show that ZFP‐1/PHA‐4 FFLs controlled the amplitude of target gene expression, duration control experiments could not be performed in the *eat‐2* mutant. However, similar to DAF‐16, we found that ZFP‐1 peaks overlapped with PHA‐4 peaks in the ChIP‐seq experiments (Fig. 5C, left panel) although not all the PHA‐4 peaks are occupied by ZFP‐1 (Fig. 5C, right panel). Finally, a significant number of genes having PHA‐4 binding sites in the 2.0 kb promoter region overlapped with targets of ZFP‐1 (*P *=* *5.54e‐133 by hypergeometric test), showing that ZFP‐1 may also fine‐tune the expression of a large number of PHA‐4 direct targets (Fig. 5D).

### ZFP‐1 and GFL‐1 are required for IIS‐ as well as DR‐mediated lifespan extension

Considering the role of DAF‐16/FOXO and PHA‐4/FOXA in regulating the amplitude/duration of their target genes using ZFP‐1/GFL‐1, knocking down the latter may deregulate a large part of the transcriptome during low IIS or DR. So we asked whether knocking down *zfp‐1/gfl‐1* will influence the longevity associated with these pathways.

**Figure 6:**
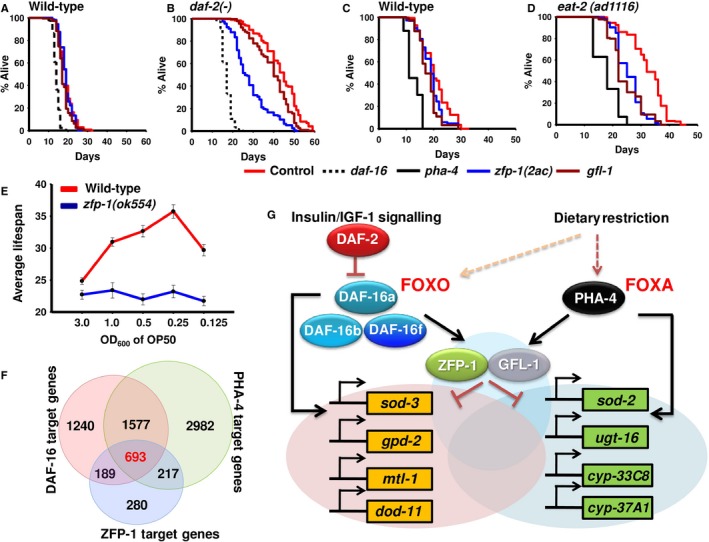
ZFP‐1 acts as a converging point for the IIS and dietary restriction (DR) pathways to regulate longevity and development. Effects of *zfp‐1(2ac)*,* gfl‐1* or *daf‐16* knockdown on lifespan of WT (A) and *daf‐2(‐)*(B). The *daf‐16* RNAi was taken as control. Effects of *zfp‐1(2ac)*,* gfl‐1* or *pha‐4* knockdown on lifespan of wild‐type (C) and *eat‐2(ad1116)* (D). The *pha‐4* RNAi was taken as control. (E) In *zfp‐1(ok554)*, bacterial dilution‐induced DR (bDR) failed to produce a typical bell‐shaped curve as seen in WT. Error bars are SEM between two experiments. Lifespans were determined at 20 °C. (F) Overlap of direct target genes of DAF‐16, PHA‐4 and ZFP‐1 as determined by ChIP‐seq analysis. ChIP‐seq data for PHA‐4 and ZFP‐1 were obtained from MODENCODE, while DAF‐16 data were generated in the laboratory (Kumar *et al*., 2015). (G) Different isoforms of DAF‐16 and PHA‐4 transcriptionally regulate *zfp‐1* and *gfl‐1*. ZFP‐1, along with GFL‐1, in turn, may fine‐tune the expression of a large number of genes downstream of the two pathways and controlled by DAF‐16 and/or PHA‐4, thereby modulating lifespan and development.

The *daf‐2(‐)* worms live more than double that of wild‐type (Kenyon *et al*., 1993). We grew the wild‐type or mutant worms on control, *zfp‐1 (2ac), gfl‐1* or *daf‐16* RNAi since hatching and performed lifespan analysis. We found that *zfp‐1(2ac)* RNAi suppressed the *daf‐2(‐)* lifespan by ~32% (Fig. 6B, Tables S1, S3). This shortening of *daf‐2(‐)* lifespan by *zfp‐1* RNAi was not simply because of sickness as the RNAi did not shorten lifespan of wild‐type, *daf‐16(‐)* or *daf‐16(‐);daf‐2(‐)* to such extent (Figs 6A, S6A and B, Tables S1, S3). The *gfl‐1* RNAi had a small but statistically significant effect on the *daf‐2(‐)* as well as WT lifespans.

The *daf‐16(a)* and *daf‐16(f)* isoforms regulate lifespan differentially with the *daf‐16(f)* as the major contributor (Kwon *et al*., 2010). We knocked down the *zfp‐1* and *gfl‐1* in *daf‐16(‐);daf‐2(‐);daf‐16(a)* or *daf‐16(‐);daf‐2(‐);daf‐16(f)* and found that *zfp‐1(2ac)* RNAi was mainly able to suppress the *daf‐16(‐);daf‐2(‐);daf‐16(f)* lifespan (Figure S6D, Tables S1, S3) with no effect on *daf‐16(‐);daf‐2(‐);daf‐16(a)* (Figure S6C). To determine whether this effect was indeed specific, we evaluated another phenotype that is controlled by IIS, that is dauer formation. Interestingly for this phenotype, although both DAF‐16 isoforms (a and f) contribute to the phenotype, *zfp‐1(2ac)* RNAi was found to enhance only the DAF‐16(a)‐mediated dauer formation (Figure S6E–G). The *gfl‐1* RNAi decreased lifespan in a small but statistically significant manner in both *daf‐16(‐);daf‐2(‐);daf‐16(a)*and *daf‐16(‐);daf‐2(‐);daf‐16(f)* (Figure S6C,D), similar to wild‐type. This suggests that in *daf‐2(‐)*,* zfp‐1(2ac)* RNAi may be suppressing the effect of *daf‐16(f)* isoform. Together, *zfp‐1* differentially affects lifespan and dauer phenotypes associated with the IIS pathway that are regulated by DAF‐16. In line with previous reports that DAF‐16 regulates longevity of germline‐ablated worms and TOR pathway mutants (Berman & Kenyon, 2006; Robida‐Stubbs *et al*., 2012), we observed that the increased lifespans of *glp‐1(e2141)* and *let‐363(ok3018)* were also partially dependent on *zfp‐1* and *gfl‐1* (Figure S7A–D).

Although *gfl‐1* is a strong target of DAF‐16, we failed to observe any dramatic effect on lifespan of the low IIS mutant. It is possible that GFL‐1 is required downstream of the IIS to regulate phenotypes other than lifespan. This was indeed true as knocking down *gfl‐1* in *daf‐2(‐)* suppressed dauer formation significantly (Figure S6E,F). Similar to *zfp‐1(2ac)*, this effect on dauer formation was due to the contribution of *daf‐16(a)* and not the *daf‐16(f)* isoform (Figure S6G). Together, ZFP‐1 and GFL‐1 are required differentially for phenotypes controlled by the IIS pathway that are dependent on different isoforms of DAF‐16.

Next, we studied the role of ZFP‐1 and GFL‐1 in DR‐mediated longevity enhancement. As mentioned earlier, DR‐like condition can be initiated either by using the *eat‐2* mutants or by knocking down *drl‐1* or else by diluting the bacterial feed, all these interventions requiring PHA‐4. Lifespan analysis showed that knocking down *zfp‐1(2ac)* or *gfl‐1* significantly decreased lifespan of *eat‐2(ad1116)*,* eat‐2(ad1113)* and *eat‐2(ad1116);rrf‐3(pk1426)* (Figs 6D, S8B, C, Tables S1, S3). We did not observe a significant suppression in the case of *eat‐2(ad465)* (Figure S8A, Tables S1, S3). This shortening was not a nonspecific effect of sickness as we did not observe similar effects in the WT or RNAi‐hypersensitive mutant *rrf‐3(pk1426)* (Figs 6A, C, and S8D). Importantly, lifespan increase observed under *drl‐1* (Chamoli *et al*., 2014) knockdown was completely dependent on *zfp‐1* (Figure S8E), but independent of *gfl‐1* (Figure S8F). For the latter case, it may also be noted that the expression of *gfl‐1* does not change significantly on *drl‐1* knockdown (Figure S4E), supporting the fact that different paradigms of DR may function through distinct mechanisms. This dependence was not due to the fact that *zfp‐1* deletion prevents RNAi from working as we found that *zfp‐1(ok554)* failed to grow on *pos‐1* RNAi, similar to WT (Figure S8G) (Dudley *et al*., 2002; Grishok *et al*., 2005). The *pos‐1* gene codes for a CCCH‐type zinc finger protein that is required for specification of germ cells, intestine, pharynx and hypodermis (Tabara *et al*., 1999); knocking it down leads to larval lethality. Finally, in *zfp‐1(ok554)*, the nongenetic mode of dietary restriction was unable to generate the typical bell‐shaped curve when average lifespan was plotted against the bacterial concentration (OD_600_), as seen in WT (Fig. 6E, Tables S1, S3). Together, these data suggest that *zfp‐1* and *gfl‐1* are also regulated by PHA‐4/FOXA and are differentially required for longevity in multiple paradigms of DR or in conditions that mimic DR.

## Discussion

### IIS and DR pathways commonly employ transcriptional feed‐forward loops involving ZFP‐1/AF10

DAF‐16/FOXO is a central regulator of longevity, stress tolerance, metabolism and development downstream of the IIS cascade (Kenyon, 2010). It is also required for longevity assurance through germline and neuronal signalling as well as for some DR regimes (Greer & Brunet, 2009; Kenyon, 2010). On the other hand, PHA‐4/FOXA that is an important factor in pharynx development (Gaudet & Mango, 2002) is also required for select DR‐induced lifespan extension (Panowski *et al*., 2007). How these transcription factors can regulate such a diverse array of phenotypes is an interesting and emerging area of research. Extensive research over the last decade has shown that DAF‐16 regulates a large number of genes, directly or indirectly (McElwee *et al*., 2003; Murphy *et al*., 2003; Oh *et al*., 2006; Schuster *et al*., 2010; Riedel *et al*., 2013; Tepper *et al*., 2013; Kumar *et al*., 2015). The activity of DAF‐16 is also regulated by multiple kinases including MST‐1, JNK‐1 and AMPK apart from the IIS pathway kinases (Oh *et al*., 2005; Lehtinen *et al*., 2006; Greer *et al*., 2007a,b; Kwon *et al*., 2010). Several modifying enzymes also interact with DAF‐16 to regulate its function under different conditions, including SIR‐2, RLE‐1, PRMT‐1 and p300/CBP‐1 (Wolff *et al*., 2006; Takahashi *et al*., 2011; Chiang *et al*., 2012; Riedel *et al*., 2013). Recently, DAF‐16 was found to employ chromatin‐modifying enzymes SWI/SNF to regulate gene expression (Riedel *et al*., 2013). On the other hand, very little is known about how PHA‐4 functions (Pandit *et al*., 2014). However, none of the regulators of these transcription factors have been reported to be a transcriptional direct target. Our study provides a glimpse of an additional level of sophistication in DAF‐16/FOXO biology and a new insight into PHA‐4 regulation. We show that DAF‐16 as well as PHA‐4 directly regulates the transcription of ZFP‐1, a protein involved in chromatin remodelling. ZFP‐1, on the other hand, modulates the transcription of DAF‐16‐ as well as PHA‐4‐regulated genes, possibly by preventing access of the transcription factors to chromatin, forming incoherent feed‐forward loops (Fig. 6G). ZFP‐1 is known to recruit DOT1.1 on actively transcribed genes to deposit H3K79 methylation, opposing H2B ubiquitination, to initiate a negative feedback regulation that modulates gene expression (Cecere *et al*., 2013). Thus, in the presence of ZFP‐1, chromosome compaction may ensue at the promoters that possibly prevent the access of DAF‐16 or PHA‐4 to the chromatin; in the absence of ZFP‐1, these transcription factors thus show a higher recruitment to the promoter of target genes.

### Isoform‐specific interaction between DAF‐16 and ZFP‐1

Our data reveal an intricate relationship between the different isoforms of DAF‐16 and ZFP‐1. Previous studies have pointed at overlapping as well as distinct functions of the DAF‐16 isoforms that differentially affect lifespan (Kwon *et al*., 2010), with the DAF‐16(f) as the major contributor. Although all the isoforms of DAF‐16 regulate the expression of the *zfp‐1* isoforms during low IIS condition, we found that *zfp‐1* knockdown only affected the enhanced lifespan of the *daf‐16(‐);daf‐2(‐);daf‐16(f)* strain and not the one rescued with DAF‐16(a) isoform. On the other hand, ZFP‐1 interacted with DAF‐16(a) for dauer regulation. Thus, ZFP‐1 may influence the recruitment of different DAF‐16 isoforms, thereby differentially affecting their downstream genes and the resulting phenotypes.

### ZFP‐1 fine‐tunes gene expression downstream of IIS and DR

The IIS and DR pathways are critical signal transducers that promptly respond to changes in nutritional status of an organism (Kenyon, 2010). Tight control of these pathways is essential for survival as evidenced by the lethality and/or arrest seen in insulin receptor‐knockout animals (Gems *et al*., 1998; Kitamura *et al*., 2003) and lifespan shortening when DR proceeds towards starvation (Mair & Dillin, 2008). Interestingly however, lowering the signals through IIS pathway or optimum nutrition can increase lifespan across species (Kenyon, 2010). Because DAF‐16/FOXO and PHA‐4/FOXA are the major outputs of these pathways, they need to be under strict regulation. While these proteins act as transcriptional activators, the system must build in safeguards to reduce the amplitude and duration of gene expression following the activation of the transcription factors by upstream kinases. One possible way of achieving that is through degradation by a ubiquitin‐proteasome pathway. But in the case of IIS, the RLE‐1‐mediated removal of DAF‐16 may have been designed to eliminate inactive DAF‐16 accumulating in the cytoplasm following AKT phosphorylation (Li *et al*., 2007). Alternatively, microRNA may be utilized effectively for this purpose. Although not known for DAF‐16/FOXO, we have recently shown that PHA‐4/FOXA may utilize microRNA FFLs to fine‐tune gene expression during DR (Pandit *et al*., 2014). By using such FFLs, the system builds in robustness that may reduce phenotypic variations. Here, we identify a mechanism that is common to both the pathways. We propose that the ZFP‐1‐mediated FFLs may have been devised to control the amplitude and duration of DAF‐16‐ or PHA‐4‐mediated gene expression, within the nucleus, following the changes in the nutrition available to the organism. By effectively coupling the activation of downstream genes with simultaneous upregulation of the negative regulator (ZFP‐1), IIS and DR pathways ensure that target genes are not overactivated. Thus, knocking down *zfp‐1* in *daf‐2(‐)* or during DR may lead to the deregulation of a large number of DAF‐16 or PHA‐4 target genes, ultimately affecting longevity and other phenotypes. This type of interaction may not be limited to IIS or DR and may be commonly employed by other signalling pathways that culminate on DAF‐16 or PHA‐4.

### ZFP‐1 represents another converging point for IIS and DR

The IIS and the DR pathways may respond to distinct nutritional cues to modulate gene expression, animal development and lifespan. While insulin signalling is typically sensitive to glucose availability, DR can be initiated even by restricting the intake of a single amino acid (Zimmerman *et al*., 2003; MacDonald *et al*., 2005; Miller *et al*., 2005; Grandison *et al*., 2009; Fontana & Partridge, 2015). Perfunctorily, it may appear that DR increases lifespan by modulating the IIS pathway, but these pathways often work independently. The transcription factor output of one of these signal transduction pathways may not always be licensed by the other, especially in the case of the IIS. These pathways, however, converge on core transcription factors like DAF‐16, SKN‐1 and HLH‐30 or coregulators like SMK‐1 to regulate DR‐ or IIS‐specific gene expression. Here, we provide new evidence that these two pathways also converge onto a conserved chromatin modifier to possibly fine‐tune the expression of an overlapping set of genes. In fact, we found that a considerable number of genes are commonly bound by DAF‐16, PHA‐4 as well as ZFP‐1 that may constitute important longevity genes (Fig. 6F). This observation raises an interesting conundrum; if such a large number of genes are shared between IIS and DR, why knocking down *daf‐16* does not affect all forms of DR or why PHA‐4 is not required for *daf‐2(e1370)* lifespan. Although both the pathways involve ZFP‐1‐mediated FFLs to regulate the expression of overlapping sets of genes, they are probably activated under different temporal or spatial windows of nutritional cues. It is also possible that the IIS and DR pathways have different thresholds and are activated independently. Nonetheless, by using a common downstream factor like ZFP‐1/AF10, the signalling cascades may have designed a possible mechanism for synergism if both cascades are activated simultaneously.

Together, our study shows the intricate nature of gene regulatory modules downstream of IIS and DR pathways that may help fine‐tune gene expression for an enhanced longevity (Fig. 6G). Due to the conserved nature of the proteins, we posit that similar mechanisms may exist in mammals.

## Materials and methods

Detailed as well as additional materials and methods are provided as a supplementary file. Unless otherwise mentioned, all strains were maintained at 20 °C using standard *C. elegans* techniques (Stiernagle, 2006). The *zfp‐1(2ac)* (752 bp) was amplified from wild‐type cDNA and cloned into the *pL4440* vector (Addgene, Cambridge, Massachusetts, USA) for RNAi. Lifespan analysis was performed as described previously (Chamoli *et al*., 2014). Lifespan graph was plotted with percentage alive on *Y*‐axis and the number of days on the *X*‐axis. Statistical analyses for survival were conducted using Mantel–Cox log‐rank test through OASIS software available at http://sbi.postech.ac.kr/oasis (Yang *et al*., 2011). Lifespans are expressed as average lifespan ± SEM consolidated for all the lifespan experiments. Consolidated lifespan data with the number of experiments (*N*) and the number of animals (*n*) are reported in Table S1 and individual lifespan experiments are recorded in Table S3. For dauer assay, *daf‐2(‐), daf‐16(‐);daf‐2(‐);daf‐16a,* or *daf‐16(‐);daf‐2(‐);daf‐16f* animals were grown at 15 °C, and following hypochlorite treatment, eggs were placed on RNAi plates. The eggs were then incubated at 22 °C for 72 h after which they were scored for dauers. Dauers were confirmed by treating the animals with 1% SDS for an hour. For promoter‐gfp reporter assays, the eggs were grown on different RNAi bacteria at 20 °C till they reached L4‐YA. The worms were photographed using Axioimager M2 (Zeiss, Oberkochen, Germany), and fluorescence intensity was quantified using NIH ImageJ software (http://imagej.nih.gov/ij/index.html). Fluorescence intensity is represented as percentage fluorescence of control RNAi. For duration control experiments, the *daf‐2(‐);Psod‐3::gfp* worms were maintained and grown at 15 °C till L2 and then shifted to 25 °C for 18 h to lower the flux in the IIS pathway. Subsequently, they were shifted back to 15 °C for 12 h before being photographed under fluorescence microscope. Fluorescence intensity is represented in arbitrary units. RNA isolation and QRT–PCR were performed as previously reported (Chamoli *et al*., 2014). ChIP was performed as described previously (Oh *et al*., 2006; Kumar *et al*., 2015).

## Conflict of interest

The authors declare no conflict of interest.

## Author contributions

AM conceived the project and wrote the manuscript with the help from AS. NK and AG performed ChIP experiments; LM generated transgenic lines; VJ analysed ChIP‐seq data along with NK; AS performed all other experiments and analysis.

## Funding

Department of Biotechnology, Ministry of Science and Technology, (Grant/Award Number: ‘BT/HRD/35/02/12/2008’, ‘BT/PR13720/BAB/10/779/2010’)

Indian Council of Medical Research, (Grant/Award Number: ‘54/3/CFP/GER/2011‐NCD‐II’).

## Supporting information


**Fig. S1** (A) Positions of SL1 (splice leader) sites in the *zfp‐1(2a) and zfp‐1(2c)* transcripts.
**Fig. S2** (A) ChIP‐PCR analysis of the binding of DAF‐16/FOXO isoforms (a‐upper, b‐middle or f‐lower panel) on the promoter of *gfl‐1*.
**Fig. S3** (A) ChIP‐PCR analysis of binding of DAF‐16/FOXO isoforms (a‐upper, b‐middle or f‐lower panel) to the different regions on the promoters of *zfp‐1(2a)* and *zfp‐1(2c)*.
**Fig. S4** (A) UCSC browser view of PHA‐4/FOXA peak on *gfl‐1* promoter as determined by reanalysis of ChIP‐seq data of OP37 strain; data mined from MODENCODE.
**Fig. S5** (A) QRT–PCR detection of mRNA levels of DAF‐16 targets in wild‐type or *daf‐2(‐)*.
**Fig. S6** (A–D) Lifespan analysis of indicated strains on control, *zfp‐1(2ac)*,* gfl‐1* or *daf‐16* RNAi.
**Fig. S7** (A–D) Lifespan analysis of indicated strains on control, *zfp‐1(2ac)*,* gfl‐1* or *daf‐16* RNAi.
**Fig. S8** (A,B) Lifespan of different *eat‐2* alleles on control, *zfp‐1(2ac)*,* gfl‐1* or *pha‐4* RNAi.

**Table S1** Details of life span experiments performed (consolidated data).

**Table S2** List of primers used in the study.

**Table S3** Details of life span experiments performed (Individual experiments used for consolidation).

**Table S4** GO term analysis of the 885 genes that are common targets of DAF‐16 and ZFP‐1 when *C. elegans* whole genome was used as a background.

**Data S1** Materials and methods.

## References

[acel12477-bib-0001] Alon U (2007) Network motifs: theory and experimental approaches. Nat. Rev. Genet. 8, 450. 17510665 10.1038/nrg2102

[acel12477-bib-0002] Avgousti DC , Cecere G , Grishok A (2013) The conserved PHD1‐PHD2 domain of ZFP‐1/AF10 is a discrete functional module essential for viability in Caenorhabditis elegans. Mol. Cell. Biol. 33, 999–1015. 23263989 10.1128/MCB.01462-12 PMC3623080

[acel12477-bib-0003] Berman JR , Kenyon C (2006) Germ‐cell loss extends *C. elegans* life span through regulation of DAF‐16 by kri‐1 and lipophilic‐hormone signaling. Cell 124, 1055–1068. 16530050 10.1016/j.cell.2006.01.039

[acel12477-bib-0004] Bishop NA , Guarente L (2007) Two neurons mediate diet‐restriction‐induced longevity in *C. elegans* . Nature 447, 545–549. 17538612 10.1038/nature05904

[acel12477-bib-0005] Caudell D , Aplan PD (2008) The role of CALM‐AF10 gene fusion in acute leukemia. Leukemia 22, 678–685. 18094714 10.1038/sj.leu.2405074 PMC2366104

[acel12477-bib-0006] Cecere G , Hoersch S , Jensen M , Dixit S , Grishok A (2013) The ZFP‐1(AF10)/DOT‐1 Complex Opposes H2B Ubiquitination to Reduce Pol II Transcription. Mol. Cell 50, 894. 23806335 10.1016/j.molcel.2013.06.002 PMC3784254

[acel12477-bib-0007] Chamoli M , Singh A , Malik Y , Mukhopadhyay A (2014) A novel kinase regulates dietary restriction‐mediated longevity in *Caenorhabditis elegans* . Aging Cell 13, 641–655. doi:10.1111/acel.12218. 24655420 10.1111/acel.12218 PMC4326946

[acel12477-bib-0008] Chiang WC , Tishkoff DX , Yang B , Wilson‐Grady J , Yu X , Mazer T , Eckersdorff M , Gygi SP , Lombard DB , Hsu AL (2012) *C. elegans* SIRT6/7 homolog SIR‐2.4 promotes DAF‐16 relocalization and function during stress. PLoS Genet. 8, e1002948. 23028355 10.1371/journal.pgen.1002948 PMC3441721

[acel12477-bib-0009] Debernardi S , Bassini A , Jones L , Chaplin T , Linder B , de Bruijn D , Meese E , Young B (2002) The MLL fusion partner AF10 binds GAS41, a protein that interacts with the human SWI/SNF complex. Blood 99, 275. 11756182 10.1182/blood.v99.1.275

[acel12477-bib-0010] Dennis G Jr , Sherman BT , Hosack DA , Yang J , Gao W , Lane HC , Lempicki RA (2003) DAVID: Database for Annotation, Visualization, and Integrated Discovery. Genome Biol. 4, P3. 12734009 PMC193660

[acel12477-bib-0011] Dudley NR , Labbe JC , Goldstein B (2002) Using RNA interference to identify genes required for RNA interference. Proc. Natl Acad. Sci. USA 99, 4191–4196. 11904378 10.1073/pnas.062605199 PMC123624

[acel12477-bib-0012] Fontana L , Partridge L (2015) Promoting health and longevity through diet: from model organisms to humans. Cell 161, 106–118. 25815989 10.1016/j.cell.2015.02.020 PMC4547605

[acel12477-bib-0013] Gaudet J , Mango SE (2002) Regulation of organogenesis by the *Caenorhabditis elegans* FoxA protein PHA‐4. Science 295, 821–825. 11823633 10.1126/science.1065175

[acel12477-bib-0014] Gems D , Sutton AJ , Sundermeyer ML , Albert PS , King KV , Edgley ML , Larsen PL , Riddle DL (1998) Two pleiotropic classes of daf‐2 mutation affect larval arrest, adult behavior, reproduction and longevity in *Caenorhabditis elegans* . Genetics 150, 129–155. 9725835 10.1093/genetics/150.1.129 PMC1460297

[acel12477-bib-0015] Grandison RC , Piper MD , Partridge L (2009) Amino‐acid imbalance explains extension of lifespan by dietary restriction in *Drosophila* . Nature 462, 1061–1064. 19956092 10.1038/nature08619 PMC2798000

[acel12477-bib-0016] Greer EL , Brunet A (2009) Different dietary restriction regimens extend lifespan by both independent and overlapping genetic pathways in *C. elegans* . Aging Cell 8, 113–127. 19239417 10.1111/j.1474-9726.2009.00459.x PMC2680339

[acel12477-bib-0017] Greer EL , Dowlatshahi D , Banko MR , Villen J , Hoang K , Blanchard D , Gygi SP , Brunet A (2007a) An AMPK‐FOXO pathway mediates longevity induced by a novel method of dietary restriction in *C. elegans* . Curr. Biol. 17, 1646–1656. 17900900 10.1016/j.cub.2007.08.047 PMC2185793

[acel12477-bib-0018] Greer EL , Oskoui PR , Banko MR , Maniar JM , Gygi MP , Gygi SP , Brunet A (2007b) The energy sensor AMP‐activated protein kinase directly regulates the mammalian FOXO3 transcription factor. J. Biol. Chem. 282, 30107–30119. 17711846 10.1074/jbc.M705325200

[acel12477-bib-0019] Grishok A , Sinskey JL , Sharp PA (2005) Transcriptional silencing of a transgene by RNAi in the soma of *C. elegans* . Genes Dev. 19, 683–696. 15741313 10.1101/gad.1247705 PMC1065722

[acel12477-bib-0020] Grishok A , Hoersch S , Sharp P (2008) RNA interference and retinoblastoma‐related genes are required for repression of endogenous siRNA targets in *Caenorhabditis elegans* . Proc. Natl Acad. Sci. USA 105, 20386. 19073934 10.1073/pnas.0810589105 PMC2629315

[acel12477-bib-0021] Kenyon C (2005) The plasticity of aging: insights from long‐lived mutants. Cell 120, 449–460. 15734678 10.1016/j.cell.2005.02.002

[acel12477-bib-0022] Kenyon CJ (2010) The genetics of ageing. Nature 464, 504–512. 20336132 10.1038/nature08980

[acel12477-bib-0023] Kenyon C , Chang J , Gensch E , Rudner A , Tabtiang R (1993) A *C. elegans* mutant that lives twice as long as wild type. Nature 366, 461–464. 8247153 10.1038/366461a0

[acel12477-bib-0024] Kitamura T , Kahn CR , Accili D (2003) Insulin receptor knockout mice. Annu. Rev. Physiol. 65, 313–332. 12471165 10.1146/annurev.physiol.65.092101.142540

[acel12477-bib-0025] Kumar N , Jain V , Singh A , Jagtap U , Verma S , Mukhopadhyay A (2015) Genome‐wide endogenous DAF‐16/FOXO recruitment dynamics during lowered insulin signalling in *C. elegans* . Oncotarget 6, 41418–41433. 26539642 10.18632/oncotarget.6282 PMC4747164

[acel12477-bib-0026] Kwon ES , Narasimhan SD , Yen K , Tissenbaum HA (2010) A new DAF‐16 isoform regulates longevity. Nature 466, 498–502. 20613724 10.1038/nature09184 PMC3109862

[acel12477-bib-0027] Lakowski B , Hekimi S (1998) The genetics of caloric restriction in *Caenorhabditis elegans* . Proc. Natl Acad. Sci. USA 95, 13091–13096. 9789046 10.1073/pnas.95.22.13091 PMC23719

[acel12477-bib-0028] Lapierre LR , De Magalhaes Filho CD , McQuary PR , Chu CC , Visvikis O , Chang JT , Gelino S , Ong B , Davis AE , Irazoqui JE , Dillin A , Hansen M (2013) The TFEB orthologue HLH‐30 regulates autophagy and modulates longevity in *Caenorhabditis elegans* . Nat. Commun. 4, 2267. 23925298 10.1038/ncomms3267 PMC3866206

[acel12477-bib-0029] Lee RY , Hench J , Ruvkun G (2001) Regulation of *C. elegans* DAF‐16 and its human ortholog FKHRL1 by the daf‐2 insulin‐like signaling pathway. Curr. Biol. 11, 1950–1957. 11747821 10.1016/s0960-9822(01)00595-4

[acel12477-bib-0030] Lehtinen MK , Yuan Z , Boag PR , Yang Y , Villen J , Becker EB , DiBacco S , de la Iglesia N , Gygi S , Blackwell TK , Bonni A (2006) A conserved MST‐FOXO signaling pathway mediates oxidative‐stress responses and extends life span. Cell 125, 987–1001. 16751106 10.1016/j.cell.2006.03.046

[acel12477-bib-0031] Li W , Gao B , Lee S‐M , Bennett K , Fang D (2007) RLE‐1, an E3 ubiquitin ligase, regulates *C. elegans* aging by catalyzing DAF‐16 polyubiquitination. Dev. Cell 12, 235. 17276341 10.1016/j.devcel.2006.12.002

[acel12477-bib-0032] Libina N , Berman JR , Kenyon C (2003) Tissue‐specific activities of *C. elegans* DAF‐16 in the regulation of lifespan. Cell 115, 489–502. 14622602 10.1016/s0092-8674(03)00889-4

[acel12477-bib-0033] Lin K , Hsin H , Libina N , Kenyon C (2001) Regulation of the *Caenorhabditis elegans* longevity protein DAF‐16 by insulin/IGF‐1 and germline signaling. Nat. Genet. 28, 139–145. 11381260 10.1038/88850

[acel12477-bib-0034] MacDonald PE , Joseph JW , Rorsman P (2005) Glucose‐sensing mechanisms in pancreatic beta‐cells. Philos. Trans. R. Soc. Lond. B Biol. Sci. 360, 2211–2225. 16321791 10.1098/rstb.2005.1762 PMC1569593

[acel12477-bib-0035] Mair W , Dillin A (2008) Aging and survival: the genetics of life span extension by dietary restriction. Annu. Rev. Biochem. 77, 727–754. 18373439 10.1146/annurev.biochem.77.061206.171059

[acel12477-bib-0036] Mangan S , Alon U (2003) Structure and function of the feed‐forward loop network motif. Proc. Natl Acad. Sci. USA 100, 11980–11985. 14530388 10.1073/pnas.2133841100 PMC218699

[acel12477-bib-0037] Mansisidor AR , Cecere G , Hoersch S , Jensen MB , Kawli T , Kennedy LM , Chavez V , Tan MW , Lieb JD , Grishok A (2011) A conserved PHD finger protein and endogenous RNAi modulate insulin signaling in *Caenorhabditis elegans* . PLoS Genet. 7, e1002299. 21980302 10.1371/journal.pgen.1002299 PMC3183084

[acel12477-bib-0038] McElwee J , Bubb K , Thomas JH (2003) Transcriptional outputs of the *Caenorhabditis elegans* forkhead protein DAF‐16. Aging Cell 2, 111–121. 12882324 10.1046/j.1474-9728.2003.00043.x

[acel12477-bib-0039] Miller RA , Buehner G , Chang Y , Harper JM , Sigler R , Smith‐Wheelock M (2005) Methionine‐deficient diet extends mouse lifespan, slows immune and lens aging, alters glucose, T4, IGF‐I and insulin levels, and increases hepatocyte MIF levels and stress resistance. Aging Cell 4, 119–125. 15924568 10.1111/j.1474-9726.2005.00152.x PMC7159399

[acel12477-bib-0040] Mukhopadhyay A , Deplancke B , Walhout AJ , Tissenbaum HA (2008) Chromatin immunoprecipitation (ChIP) coupled to detection by quantitative real‐time PCR to study transcription factor binding to DNA in *Caenorhabditis elegans* . Nat. Protoc. 3, 698–709. 18388953 10.1038/nprot.2008.38 PMC2681100

[acel12477-bib-0041] Murphy CT , McCarroll SA , Bargmann CI , Fraser A , Kamath RS , Ahringer J , Li H , Kenyon C (2003) Genes that act downstream of DAF‐16 to influence the lifespan of *Caenorhabditis elegans* . Nature 424, 277–283. 12845331 10.1038/nature01789

[acel12477-bib-0042] Oh SW , Mukhopadhyay A , Svrzikapa N , Jiang F , Davis RJ , Tissenbaum HA (2005) JNK regulates lifespan in *Caenorhabditis elegans* by modulating nuclear translocation of forkhead transcription factor/DAF‐16. Proc. Natl Acad. Sci. USA 102, 4494–4499. 15767565 10.1073/pnas.0500749102 PMC555525

[acel12477-bib-0043] Oh SW , Mukhopadhyay A , Dixit BL , Raha T , Green MR , Tissenbaum HA (2006) Identification of direct DAF‐16 targets controlling longevity, metabolism and diapause by chromatin immunoprecipitation. Nat. Genet. 38, 251–257. 16380712 10.1038/ng1723

[acel12477-bib-0044] Pandit A , Jain V , Kumar N , Mukhopadhyay A (2014) PHA‐4/FOXA‐regulated microRNA feed forward loops during *Caenorhabditis elegans* dietary restriction. Aging 6, 1–21. 25504288 10.18632/aging.100697 PMC4247386

[acel12477-bib-0045] Panowski SH , Wolff S , Aguilaniu H , Durieux J , Dillin A (2007) PHA‐4/Foxa mediates diet‐restriction‐induced longevity of *C. elegans* . Nature 447, 550–555. 17476212 10.1038/nature05837

[acel12477-bib-0046] Riedel C , Dowen R , Lourenco G , Kirienko N , Heimbucher T , West J , Bowman S , Kingston R , Dillin A , Asara J , Ruvkun G (2013) DAF‐16 employs the chromatin remodeller SWI/SNF to promote stress resistance and longevity. Nat. Cell Biol. 15, 491. 23604319 10.1038/ncb2720 PMC3748955

[acel12477-bib-0047] Robida‐Stubbs S , Glover‐Cutter K , Lamming DW , Mizunuma M , Narasimhan SD , Neumann‐Haefelin E , Sabatini DM , Blackwell TK (2012) TOR signaling and rapamycin influence longevity by regulating SKN‐1/Nrf and DAF‐16/FoxO. Cell Metab. 15, 713–724. 22560223 10.1016/j.cmet.2012.04.007 PMC3348514

[acel12477-bib-0048] Schuster E , McElwee JJ , Tullet JM , Doonan R , Matthijssens F , Reece‐Hoyes JS , Hope IA , Vanfleteren JR , Thornton JM , Gems D (2010) DamID in *C. elegans* reveals longevity‐associated targets of DAF‐16/FoxO. Mol. Syst. Biol. 6, 399. 20706209 10.1038/msb.2010.54 PMC2950082

[acel12477-bib-0049] Stiernagle T (2006) Maintenance of *C. elegans* . WormBook, 11, 1–11. 18050451 10.1895/wormbook.1.101.1 PMC4781397

[acel12477-bib-0050] Tabara H , Hill RJ , Mello CC , Priess JR , Kohara Y (1999) pos‐1 encodes a cytoplasmic zinc‐finger protein essential for germline specification in *C. elegans* . Development 126, 1–11. 9834181 10.1242/dev.126.1.1

[acel12477-bib-0051] Takahashi Y , Daitoku H , Hirota K , Tamiya H , Yokoyama A , Kako K , Nagashima Y , Nakamura A , Shimada T , Watanabe S , Yamagata K , Yasuda K , Ishii N , Fukamizu A (2011) Asymmetric arginine dimethylation determines life span in *C. elegans* by regulating forkhead transcription factor DAF‐16. Cell Metab. 13, 505–516. 21531333 10.1016/j.cmet.2011.03.017

[acel12477-bib-0052] Tepper RG , Ashraf J , Kaletsky R , Kleemann G , Murphy CT , Bussemaker HJ (2013) PQM‐1 complements DAF‐16 as a key transcriptional regulator of DAF‐2‐mediated development and longevity. Cell 154, 676–690. 23911329 10.1016/j.cell.2013.07.006 PMC3763726

[acel12477-bib-0053] Tullet JM , Hertweck M , An JH , Baker J , Hwang JY , Liu S , Oliveira RP , Baumeister R , Blackwell TK (2008) Direct inhibition of the longevity‐promoting factor SKN‐1 by insulin‐like signaling in *C. elegans* . Cell 132, 1025–1038. 18358814 10.1016/j.cell.2008.01.030 PMC2367249

[acel12477-bib-0054] Wolff S , Ma H , Burch D , Maciel GA , Hunter T , Dillin A (2006) SMK‐1, an essential regulator of DAF‐16‐mediated longevity. Cell 124, 1039–1053. 16530049 10.1016/j.cell.2005.12.042

[acel12477-bib-0055] Yang JS , Nam HJ , Seo M , Han SK , Choi Y , Nam HG , Lee SJ , Kim S (2011) OASIS: online application for the survival analysis of lifespan assays performed in aging research. PLoS ONE 6, e23525. 21858155 10.1371/journal.pone.0023525 PMC3156233

[acel12477-bib-0056] Zhang P , Judy M , Lee SJ , Kenyon C (2013) Direct and indirect gene regulation by a life‐extending FOXO protein in *C. elegans*: roles for GATA factors and lipid gene regulators. Cell Metab. 17, 85–100. 23312285 10.1016/j.cmet.2012.12.013 PMC3969420

[acel12477-bib-0057] Zhong M , Niu W , Lu ZJ , Sarov M , Murray JI , Janette J , Raha D , Sheaffer KL , Lam HY , Preston E , Slightham C , Hillier LW , Brock T , Agarwal A , Auerbach R , Hyman AA , Gerstein M , Mango SE , Kim SK , Waterston RH , Reinke V , Snyder M (2010) Genome‐wide identification of binding sites defines distinct functions for *Caenorhabditis elegans* PHA‐4/FOXA in development and environmental response. PLoS Genet. 6, e1000848. 20174564 10.1371/journal.pgen.1000848 PMC2824807

[acel12477-bib-0058] Zimmerman JA , Malloy V , Krajcik R , Orentreich N (2003) Nutritional control of aging. Exp. Gerontol. 38, 47–52. 12543260 10.1016/s0531-5565(02)00149-3

