# Supplementary material for: A chromatin modifier integrates insulin/IGF‐1 signalling and dietary restriction to regulate longevity
Source: Aging Cell. 2016 Apr 2;15(4):694–705. doi: 10.1111/acel.12477 (PMC4933660; doi:10.1111/acel.12477)
Supplement: Supplementary file 1 — Fig. S1 (A) Positions of SL1 (splice leader) sites in the zfp‐1(2a) and zfp‐1(2c) transcripts. Fig. S2 (A) ChIP‐PCR analysis of the binding of DAF‐16/FOXO isoforms (a‐upper, b‐middle or f‐lower panel) on the promoter of gfl‐1. Fig. S3 (A) ChIP‐PCR analysis of binding of DAF‐16/FOXO isoforms (a‐upper, b‐middle or f‐lower panel) to the different regions on the promoters of zfp‐1(2a) and zfp‐1(2c). Fig. S4 (A) UCSC browser view of PHA‐4/FOXA peak on gfl‐1 promoter as determined by reanalysis of ChIP‐seq data of OP37 strain; data mined from MODENCODE. Fig. S5 (A) QRT–PCR detection of mRNA levels of DAF‐16 targets in wild‐type or daf‐2(‐). Fig. S6 (A–D) Lifespan analysis of indicated strains on control, zfp‐1(2ac), gfl‐1 or daf‐16 RNAi. Fig. S7 (A–D) Lifespan analysis of indicated strains on control, zfp‐1(2ac), gfl‐1 or daf‐16 RNAi. Fig. S8 (A,B) Lifespan of different eat‐2 alleles on control, zfp‐1(2ac), gfl‐1 or pha‐4 RNAi. [file ACEL-15-694-s001.ppt]

## Slide 1
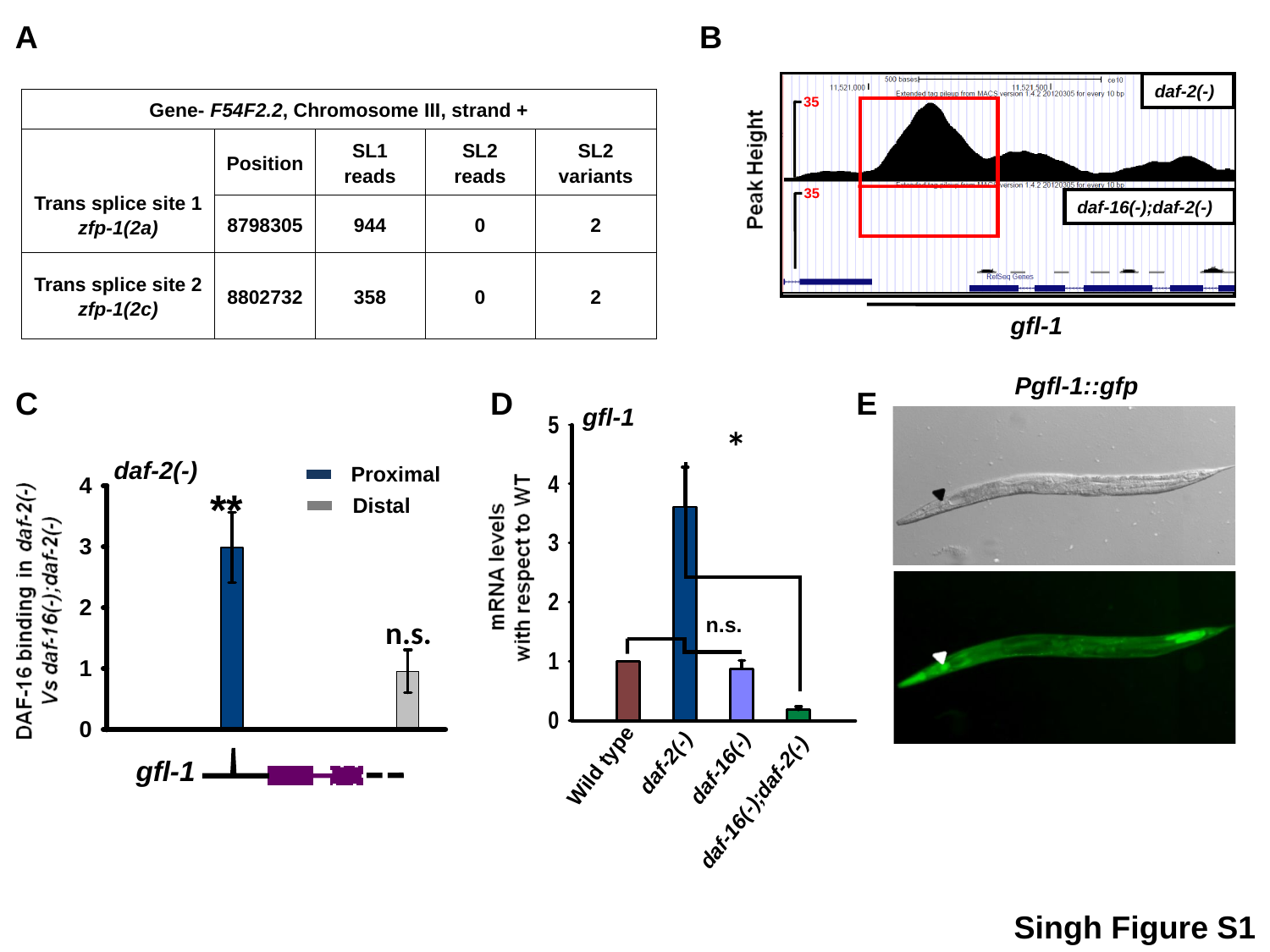

A
B
daf-2(-)
35
| Gene- F54F2.2, Chromosome III, strand + | | | | |
| --- | --- | --- | --- | --- |
| Trans splice site 1 zfp-1(2a) | Position | SL1 reads | SL2 reads | SL2 variants |
| | 8798305 | 944 | 0 | 2 |
| Trans splice site 2 zfp-1(2c) | 8802732 | 358 | 0 | 2 |
35
daf-16(-);daf-2(-)
gfl-1
Pgfl-1::gfp
C
D
E
gfl-1
*
 daf-2(-)
Proximal
**
Distal
n.s.
n.s.
daf-2(-)
Wild type
gfl-1
daf-16(-)
daf-16(-);daf-2(-)
Singh Figure S1

## Slide 2
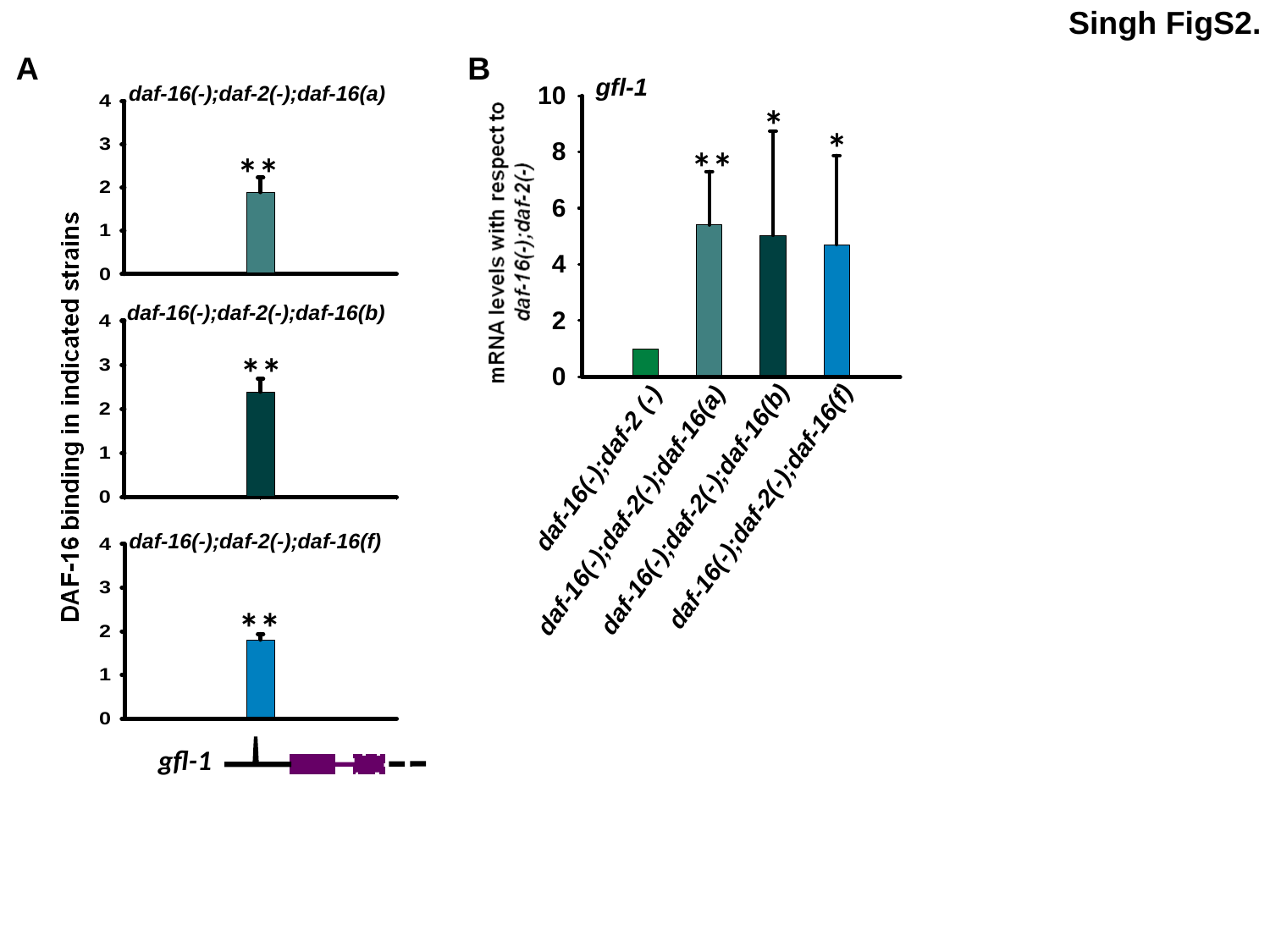

Singh FigS2.
A
B
gfl-1
 daf-16(-);daf-2(-);daf-16(a)
**
**
**
*
*
**
daf-16(-);daf-2(-);daf-16(b)
daf-16(-);daf-2 (-)
daf-16(-);daf-2(-);daf-16(f)
daf-16(-);daf-2(-);daf-16(b)
daf-16(-);daf-2(-);daf-16(a)
 daf-16(-);daf-2(-);daf-16(f)
gfl-1

## Slide 3
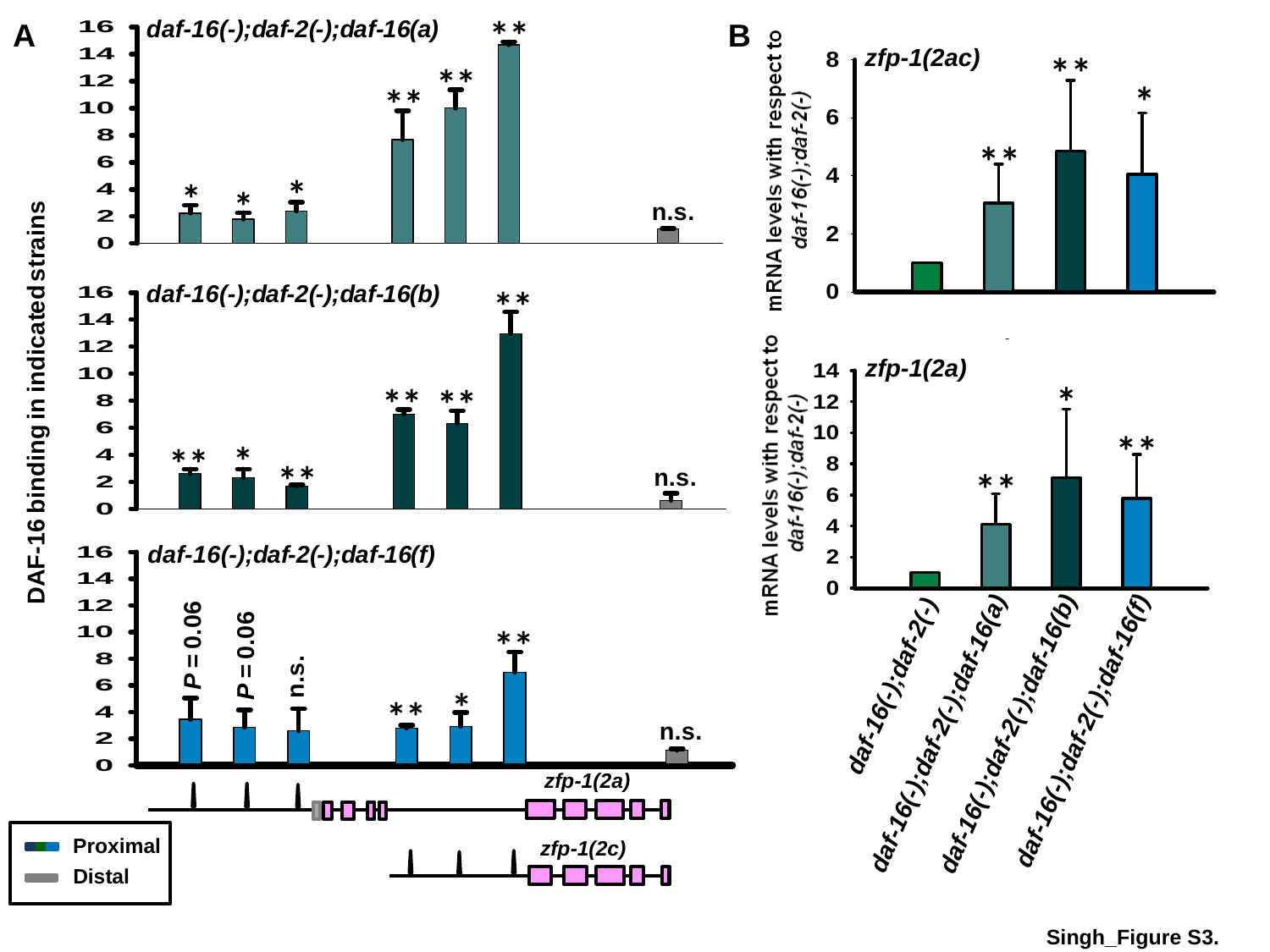

A
B
zfp-1(2ac)
**
*
**
zfp-1(2a)
*
**
**
daf-16(-);daf-2(-)
daf-16(-);daf-2(-);daf-16(f)
daf-16(-);daf-2(-);daf-16(a)
daf-16(-);daf-2(-);daf-16(b)
Singh_Figure S3.

## Slide 4
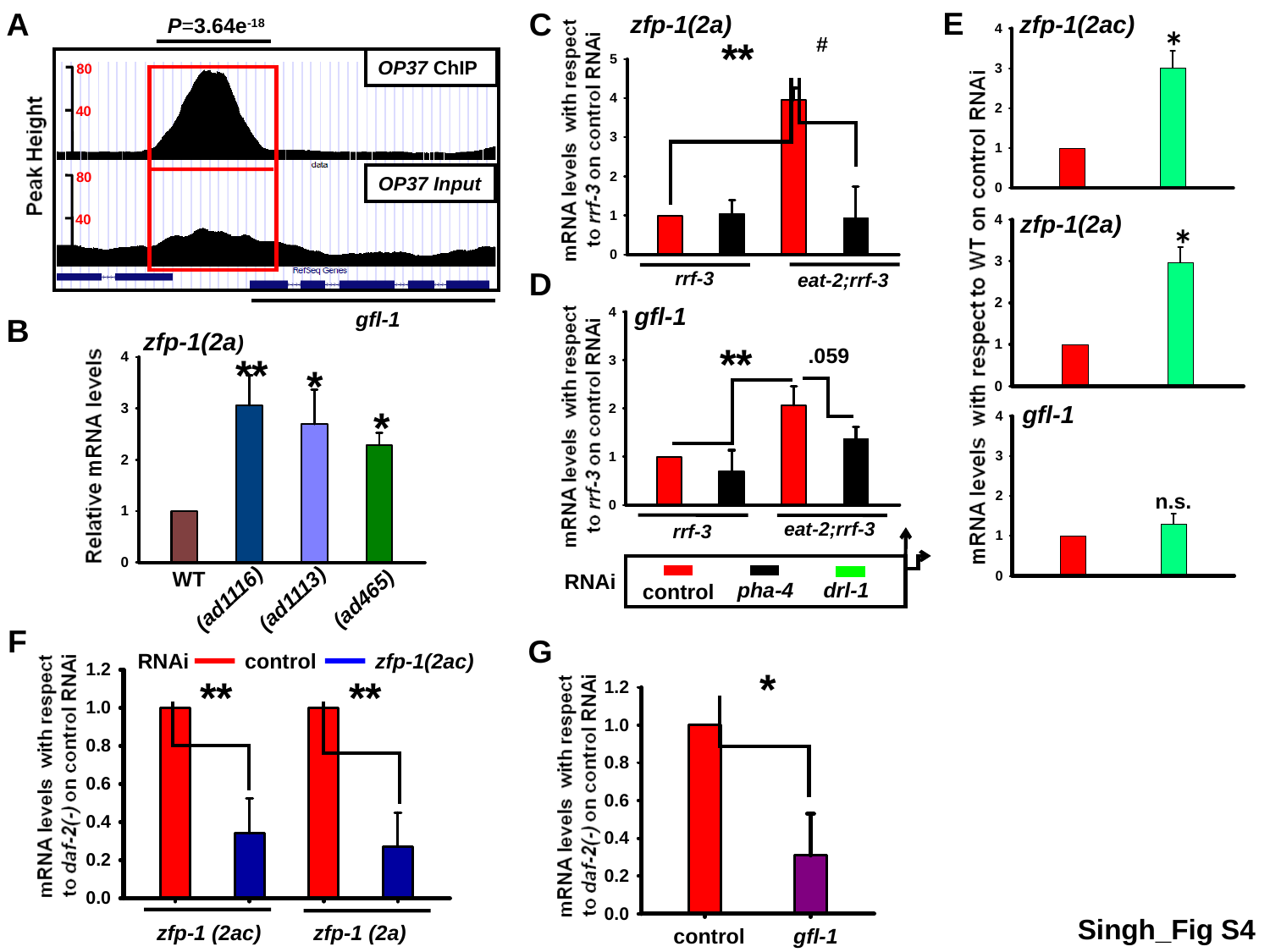

E
A
C
zfp-1(2a)
zfp-1(2ac)
P=3.64e-18
*
#
**
OP37 ChIP
80
40
80
OP37 Input
gfl-1
zfp-1(2a)
40
*
D
rrf-3
eat-2;rrf-3
gfl-1
gfl-1
B
zfp-1(2a)
**
.059
**
*
gfl-1
*
n.s.
eat-2;rrf-3
rrf-3
WT
RNAi
pha-4
drl-1
control
(ad1116)
(ad1113)
(ad465)
F
G
RNAi
 control
 zfp-1(2ac)
*
**
**
Singh_Fig S4
zfp-1 (2ac)
 zfp-1 (2a)
 control
 gfl-1

## Slide 5
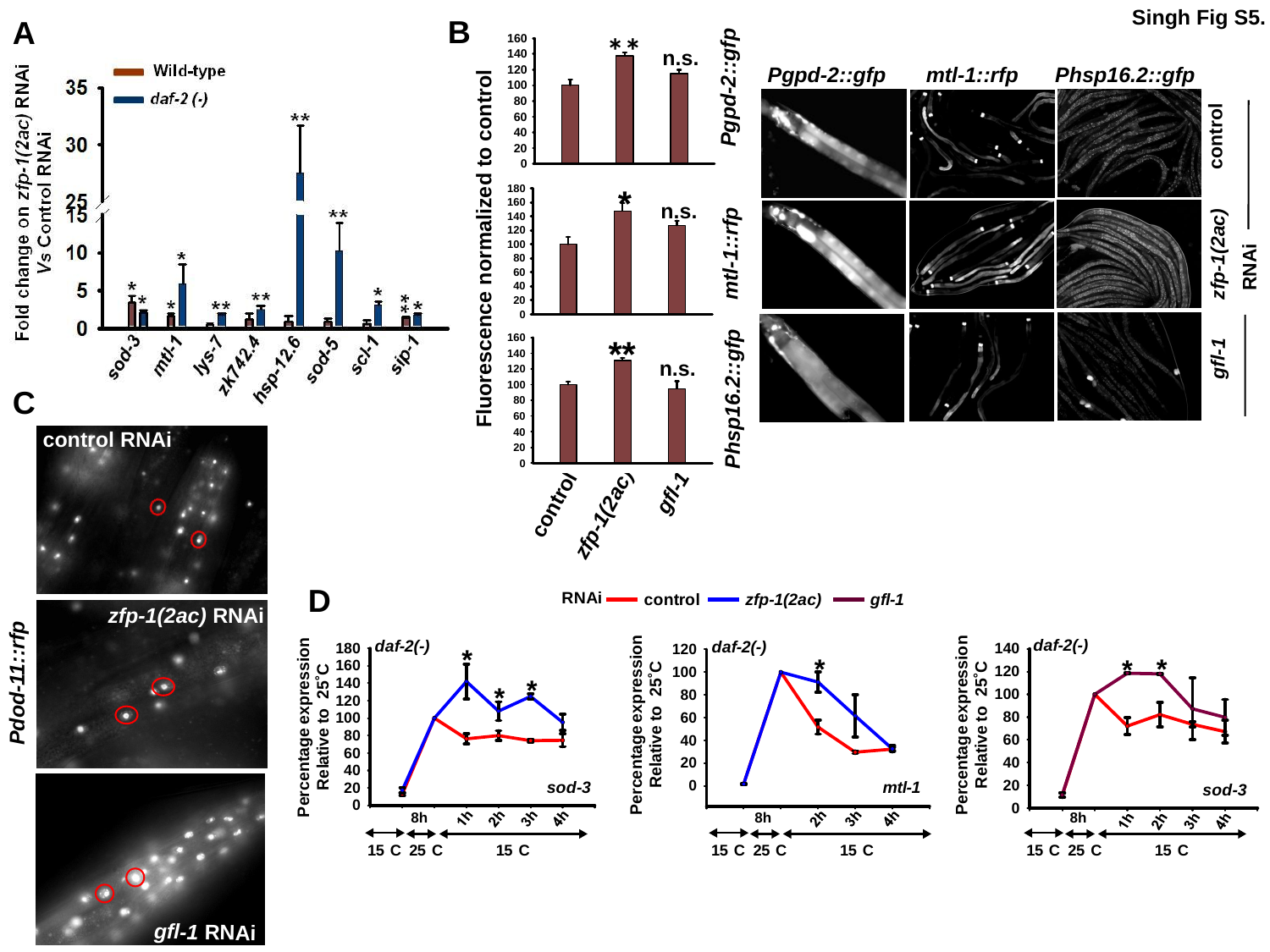

Singh Fig S5.
B
A
**
n.s.
Pgpd-2::gfp
mtl-1::rfp
Phsp16.2::gfp
Pgpd-2::gfp
 control
*
n.s.
 RNAi
Fluorescence normalized to control
mtl-1::rfp
 zfp-1(2ac)
**
 gfl-1
n.s.
C
Phsp16.2::gfp
 control RNAi
 gfl-1
 control
 zfp-1(2ac)
D
 zfp-1(2ac) RNAi
Pdod-11::rfp
 gfl-1 RNAi

## Slide 6
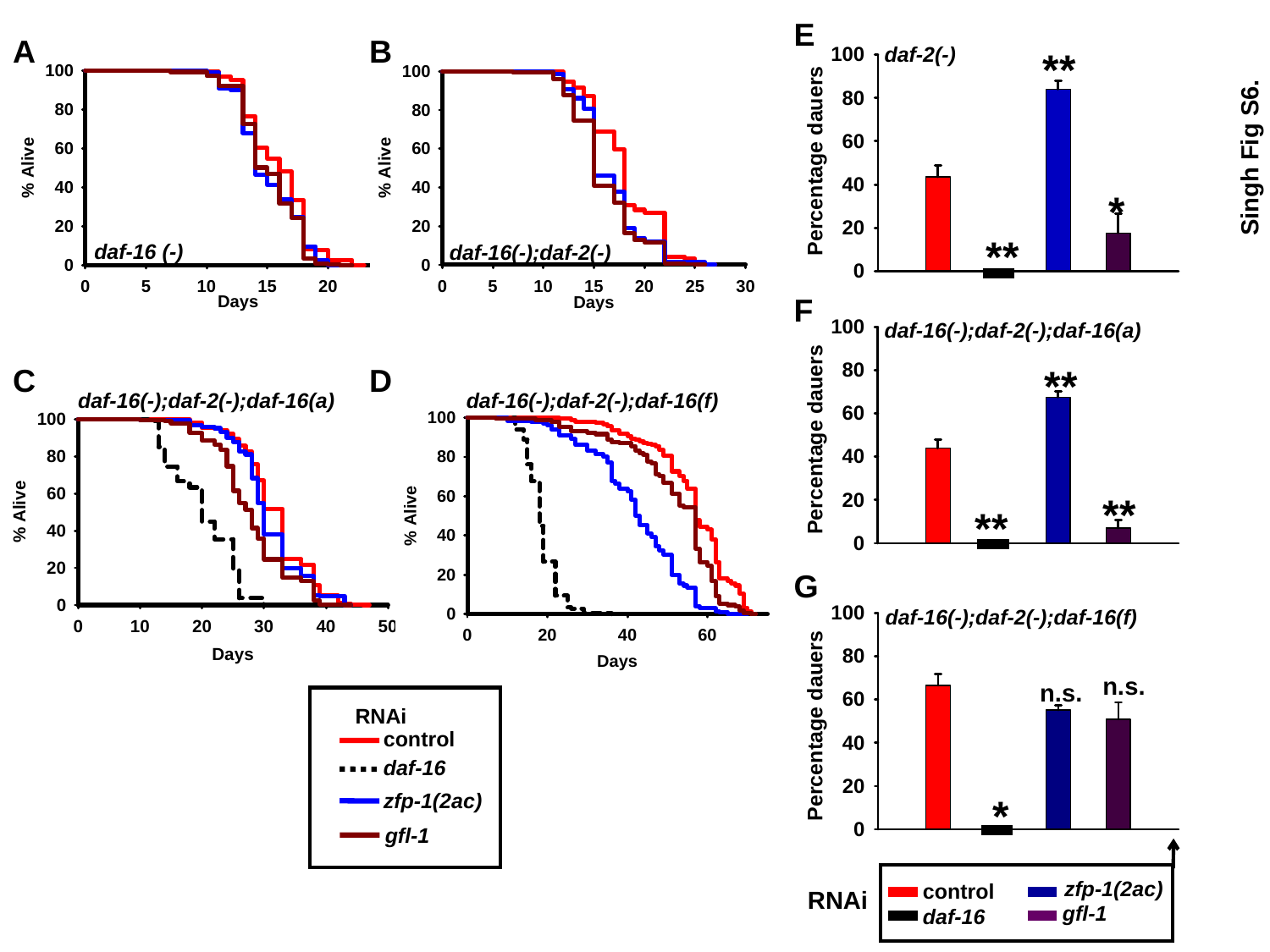

E
A
B
daf-2(-)
**
Singh Fig S6.
Percentage dauers
*
**
daf-16 (-)
daf-16(-);daf-2(-)
F
daf-16(-);daf-2(-);daf-16(a)
**
C
D
daf-16(-);daf-2(-);daf-16(a)
daf-16(-);daf-2(-);daf-16(f)
Percentage dauers
**
**
G
daf-16(-);daf-2(-);daf-16(f)
n.s.
n.s.
 RNAi
control
daf-16
zfp-1(2ac)
gfl-1
Percentage dauers
*
zfp-1(2ac)
control
daf-16
RNAi
gfl-1

## Slide 7
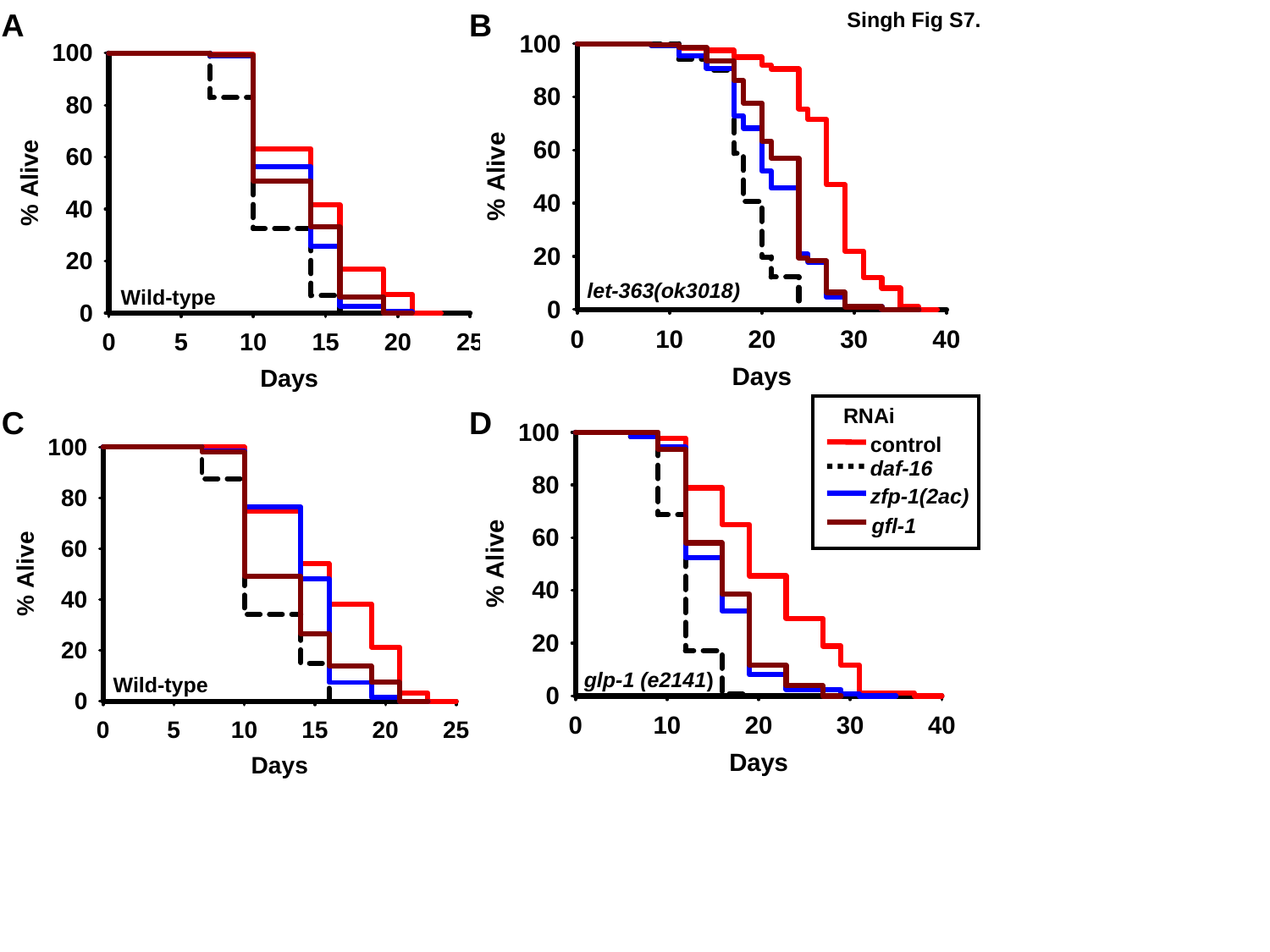

Singh Fig S7.
A
B
let-363(ok3018)
Wild-type
 RNAi
control
daf-16
zfp-1(2ac)
gfl-1
C
D
glp-1 (e2141)
Wild-type

## Slide 8
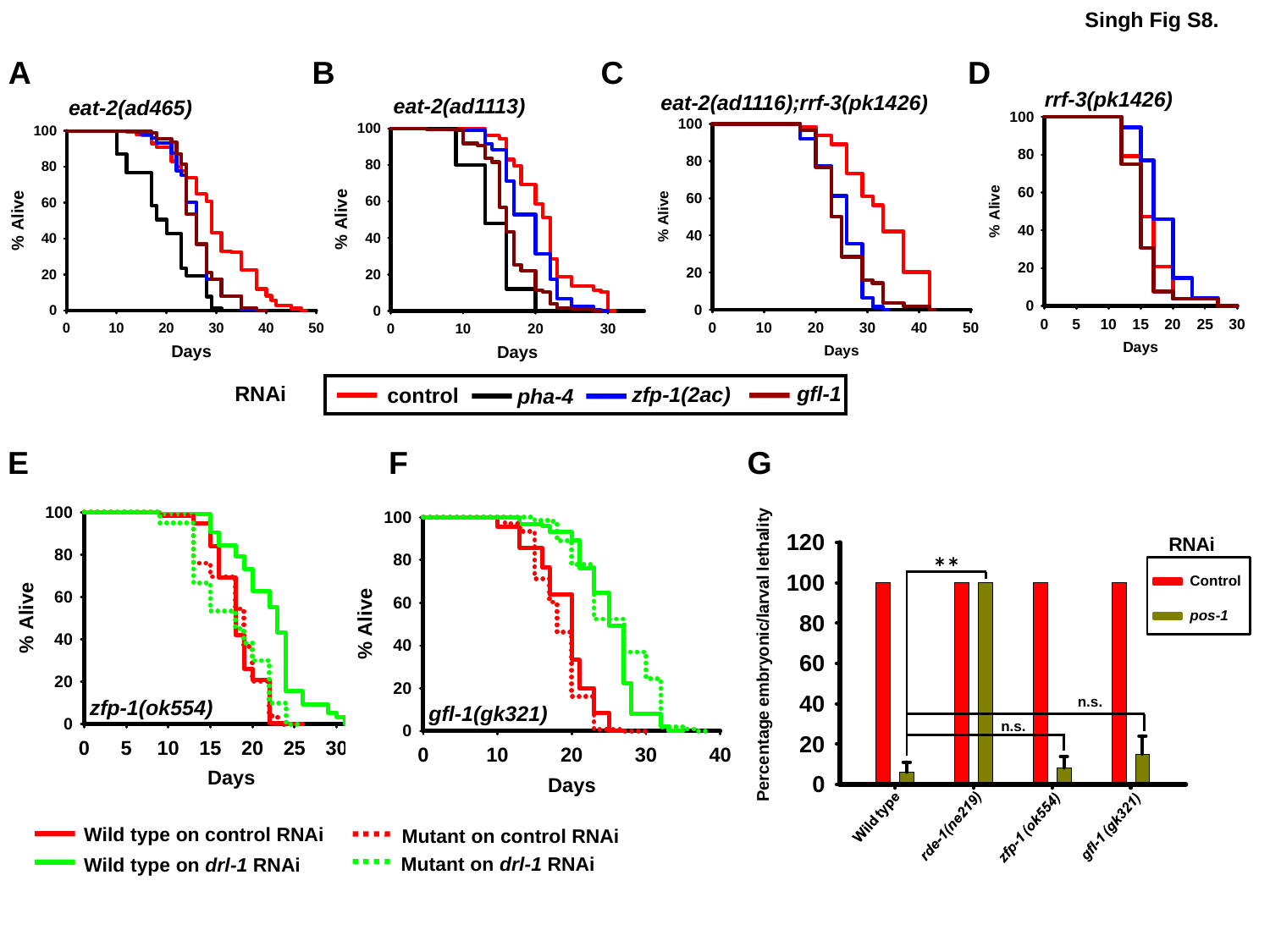

Singh Fig S8.
A
B
C
D
rrf-3(pk1426)
eat-2(ad1116);rrf-3(pk1426)
eat-2(ad1113)
eat-2(ad465)
 RNAi
 gfl-1
zfp-1(2ac)
 control
pha-4
E
F
G
zfp-1(ok554)
gfl-1(gk321)
Wild type on control RNAi
Mutant on control RNAi
Mutant on drl-1 RNAi
Wild type on drl-1 RNAi
