# Supplementary material for: A chromatin modifier integrates insulin/IGF‐1 signalling and dietary restriction to regulate longevity
Source: Aging Cell. 2016 Apr 2;15(4):694–705. doi: 10.1111/acel.12477 (PMC4933660; doi:10.1111/acel.12477)
Supplement: Supplementary file 2 — Table S1 Details of life span experiments performed (consolidated data). [file ACEL-15-694-s002.docx]

**Table S1: Details of life span experiments performed (consolidated data).**

| **Genetic Background** | **RNAi** | **Mean ± SEM** | **Percentage increase(+) or decrease (-) w.r.t. control** | **n (Number of animals)** | ***P-*value** | **N**  **(No of Experiments)** |
| --- | --- | --- | --- | --- | --- | --- |
| **Wild-type** | **control** | **19.98 ± 0.13** |  | **757** |  | **7** |
|  | ***daf-16*** | **15.01 ± 0.08** | **(-) 24.87** | **393** | **≤0.0001** | **7** |
|  | ***zfp-1(2ac)*** | **19.91 ± 0.15** | **(-) 00.35** | **378** | **0.1749** | **7** |
|  | ***gfl-1*** | **18.70 ± 0.15** | **(-) 06.41** | **412** | **≤0.0001** | **6** |
|  |  |  |  |  |  |  |
| ***daf-2(e1370)*** | **control** | **44.29 ± 0.53** |  | **311** |  | **4** |
|  | ***daf-16*** | **18.32 ± 0.14** | **(-) 58.64** | **248** | **≤0.0001** | **4** |
|  | ***zfp-1(2ac)*** | **29.82 ± 0.72** | **(-) 32.67** | **160** | **≤0.0001** | **4** |
|  | ***gfl-1*** | **40.34 ± 0.50** | **(-) 08.92** | **308** | **≤0.0001** | **4** |
|  |  |  |  |  |  |  |
| ***daf-16(mgDf50)*** | **control** | **16.85 ± 0.15** |  | **300** |  | **4** |
|  | ***zfp-1(2ac)*** | **16.06 ± 0.17** | **(-) 4.69** | **208** | **0.0012** | **4** |
|  | ***gfl-1*** | **16.11 ± 0.14** | **(-) 4.39** | **285** | **≤0.0001** | **4** |
|  |  |  |  |  |  |  |
| ***daf-16(mgDf50);daf-2(e1370)*** | **control** | **18.97 ± 0.18** |  | **352** |  | **4** |
|  | ***zfp-1(2ac)*** | **17.49 ± 0.20** | **(-) 07.80** | **224** | **≤0.0001** | **4** |
|  | ***gfl-1*** | **17.00 ± 0.20** | **(-) 10.38** | **244** | **≤0.0001** | **4** |
|  |  |  |  |  |  |  |
| ***daf-16(mgDf50);daf-2(e1370);daf-16(a)*** | **control** | **32.70 ± 0.32** |  | **280** |  | **4** |
|  | ***daf-16*** | **21.23 ± 0.33** | **(-) 35.08** | **243** | **≤0.0001** | **4** |
|  | ***zfp-1(2ac)*** | **31.66 ± 0.30** | **(-) 03.18** | **313** | **0.0221** | **4** |
|  | ***gfl-1*** | **28.93 ± 0.35** | **(-) 11.53** | **273** | **≤0.0001** | **4** |
|  |  |  |  |  |  |  |
| ***daf-16(mgDf50);daf-2(e1370);daf-16(f)*** | **control** | **57.58 ± 0.63** |  | **249** |  | **4** |
|  | ***daf-16*** | **19.56 ± 0.26** | **(-) 66.03** | **214** | **≤0.0001** | **4** |
|  | ***zfp-1(2ac)*** | **43.47 ± 0.63** | **(-) 24.51** | **311** | **≤0.0001** | **4** |
|  | ***gfl-1*** | **53.19 ± 0.68** | **(-) 07.62** | **264** | **≤0.0001** | **4** |
|  |  |  |  |  |  |  |
| **Wild-type (for *let-363*)** | **control** | **16.98 ± 0.19** |  | **247** |  | **3** |
|  | ***daf-16*** | **14.17 ± 0.15** | **(-) 16.55** | **240** | **≤0.0001** | **3** |
|  | ***zfp-1(2ac)*** | **15.92 ± 0.16** | **(-) 06.24** | **183** | **≤0.0001** | **3** |
|  | ***gfl-1*** | **16.10 ± 0.16** | **(-) 05.18** | **242** | **≤0.0001** | **3** |
|  |  |  |  |  |  |  |
| ***let-363 (ok3018)*** | **control** | **29.17 ± 0.34** |  | **200** |  | **3** |
|  | ***daf-16*** | **20.02 ± 0.22** | **(-) 31.37** | **172** | **≤0.0001** | **3** |
|  | ***zfp-1(2ac)*** | **22.84 ± 0.36** | **(-) 21.70** | **173** | **≤0.0001** | **3** |
|  | ***gfl-1*** | **23.79 ± 0.29** | **(-) 18.44** | **202** | **≤0.0001** | **3** |
|  |  |  |  |  |  |  |
| **Wild-type (for *glp-1*)¶** | **control** | **18.38 ± 0.26** |  | **183** |  | **2** |
|  | ***daf-16*** | **14.63 ± 0.19** | **(-) 20.40** | **161** | **≤0.0001** | **2** |
|  | ***zfp-1(2ac)*** | **17.09 ± 0.17** | **(-) 07.02** | **204** | **≤0.0001** | **2** |
|  | ***gfl-1*** | **16.13 ± 0.21** | **(-) 12.24** | **203** | **≤0.0001** | **2** |
|  |  |  |  |  |  |  |
| ***glp-1 (e2141)* ¶** | **control** | **24.19 ± 0.48** |  | **180** |  | **2** |
|  | ***daf-16*** | **15.25 ± 0.23** | **(-) 36.96** | **128** | **≤0.0001** | **2** |
|  | ***zfp-1(2ac)*** | **19.04 ± 0.39** | **(-) 21.29** | **124** | **≤0.0001** | **2** |
|  | ***gfl-1*** | **19.57 ± 0.35** | **(-) 19.09** | **155** | **≤0.0001** | **2** |
|  |  |  |  |  |  |  |
| **Wild-type (for *eat* mutants)** | **control** | **21.85 ± 0.31** |  | **290** |  | **3** |
|  | ***pha-4*** | **15.45 ± 0.47** | **(-) 29.29** | **175** | **≤0.0001** | **3** |
|  | ***zfp-1(2ac)*** | **20.21 ± 0.25** | **(-) 07.50** | **258** | **≤0.0001** | **3** |
|  | ***gfl-1*** | **19.11 ± 0.26** | **(-) 12.54** | **192** | **≤0.0001** | **3** |
|  |  |  |  |  |  |  |
| ***eat-2(ad1116)*** | **control** | **34.13 ± 0.46** |  | **200** |  | **3** |
|  | ***pha-4*** | **19.96 ± 0.96** | **(-) 41.52** | **158** | **≤0.0001** | **3** |
|  | ***zfp-1(2ac)*** | **27.59 ± 0.31** | **(-) 19.16** | **196** | **≤0.0001** | **3** |
|  | ***gfl-1*** | **26.07 ± 0.41** | **(-) 23.61** | **179** | **≤0.0001** | **3** |
|  |  |  |  |  |  |  |
| ***eat-2(ad465)*** | **control** | **31.57 ± 0.50** |  | **242** |  | **3** |
|  | ***pha-4*** | **21.03 ± 0.68** | **(-) 33.39** | **167** | **≤0.0001** | **3** |
|  | ***zfp-1(2ac)*** | **27.64 ± 0.31** | **(-) 12.45** | **234** | **≤0.0001** | **3** |
|  | ***gfl-1*** | **28.01 ± 0.30** | **(-) 11.28** | **211** | **≤0.0001** | **3** |
|  |  |  |  |  |  |  |
| ***eat-2(ad1113)*** | **control** | **23.51 ± 0.38** |  | **182** |  | **3** |
|  | ***pha-4*** | **15.34 ± 0.28** | **(-) 34.75** | **130** | **≤0.0001** | **3** |
|  | ***zfp-1(2ac)*** | **20.16 ± 0.36** | **(-) 14.25** | **121** | **≤0.0001** | **2** |
|  | ***gfl-1*** | **17.55 ± 0.25** | **(-) 25.35** | **185** | **≤0.0001** | **3** |
|  |  |  |  |  |  |  |
| ***rrf-3(pk1426)*** | **control** | **18.92 ± 0.50** |  | **53** |  | **1** |
|  | ***zfp-1(2ac)*** | **21.34 ± 0.44** | **(+) 12.79** | **74** | **0.0007** | **1** |
|  | ***gfl-1*** | **17.96 ± 0.46** | **(-) 05.07** | **52** | **0.1054** | **1** |
|  |  |  |  |  |  |  |
| ***eat-2(ad1116);rrf-3(pk1426)*** | **control** | **33.69 ± 0.85** |  | **64** |  | **1** |
|  | ***zfp-1(2ac)*** | **26.15 ± 0.53** | **(-) 22.38** | **62** | **≤0.0001** | **1** |
|  | ***gfl-1*** | **26.18 ± 0.67** | **(-) 22.29** | **56** | **≤0.0001** | **1** |
|  |  |  |  |  |  |  |
| **Wild-type** | **control** | **19.43 ± 0.20** |  | **169** |  | **2** |
|  | ***drl-1*** | **23.60 ± 0.41** | **(+) 21.46** | **116** | **≤0.0001** | **2** |
|  |  |  |  |  |  |  |
| ***zfp-1(ok554)*** | **control** | **19.46 ± 0.34** |  | **79** |  | **2** |
|  | ***drl-1*** | **19.20 ± 0.52** | **(-) 01.34** | **60** | **0.3787** | **2** |
|  |  |  |  |  |  |  |
| **Wild-type** | **control** | **20.89 ± 0.27** |  | **174** |  | **2** |
|  | ***drl-1*** | **26.89 ± 0.29** | **(+) 28.72** | **215** | **≤0.0001** | **2** |
|  |  |  |  |  |  |  |
| ***gfl-1(gk321)*** | **control** | **20.79 ± 0.33** |  | **136** |  | **2** |
|  | ***drl-1*** | **28.83 ± 0.35** | **(+) 38.67** | **208** | **≤0.0001** | **2** |
|  |  |  |  |  |  |  |
| **BDR Lifespan** | **OD of OP50** |  |  |  |  |  |
| **Wild-type** | **3.0** | **25.23 ± 0.43** |  | **124** |  | **3** |
|  | **1.0** | **31.25 ± 0.54** | **(+) 23.86** | **128** | **≤0.0001** | **3** |
|  | **0.5** | **33.37 ± 0.64** | **(+) 32.26** | **131** | **≤0.0001** | **3** |
|  | **0.25** | **35.54 ± 0.77** | **(+) 40.86** | **126** | **≤0.0001** | **3** |
|  | **0.125** | **30.51 ± 0.69** | **(+) 20.93** | **137** | **≤0.0001** | **3** |
|  |  |  |  |  |  |  |
| ***zfp-1(ok554)*** | **3.0** | **22.08 ± 0.67** |  | **99** |  | **3** |
|  | **1.0** | **25.91 ± 0.99** | **(+) 17.35** | **86** | **≤0.0001** | **3** |
|  | **0.5** | **24.44 ± 0.87** | **(+) 10.69** | **105** | **0.0017** | **3** |
|  | **0.25** | **25.80 ± 0.89** | **(+) 16.85** | **111** | **≤0.0001** | **3** |
|  | **0.125** | **24.97 ± 0.85** | **(+) 13.09** | **110** | **0.0008** | **3** |
| **BDR Lifespan** |  |  |  |  |  |  |
| **Wild-type** | **3.0** | **24.84 ± 0.52** |  | **85** |  | **2** |
|  | **1.0** | **30.95 ± 0.67** | **(+) 24.59** | **85** | **≤0.0001** | **2** |
|  | **0.5** | **32.64 ± 0.88** | **(+) 31.40** | **84** | **≤0.0001** | **2** |
|  | **0.25** | **35.73 ± 1.05** | **(+) 43.84** | **82** | **≤0.0001** | **2** |
|  | **0.125** | **29.67 ± 0.90** | **(+) 19.44** | **89** | **≤0.0001** | **2** |
|  |  |  |  |  |  |  |
| ***zfp-1(ok554)*** | **3.0** | **22.71 ± 0.71** |  | **65** |  | **2** |
|  | **1.0** | **23.40 ± 1.20** | **(+) 03.04** | **48** | **0.1521** | **2** |
|  | **0.5** | **21.97 ± 0.87** | **(-) 03.26** | **68** | **0.9789** | **2** |
|  | **0.25** | **23.20 ± 0.97** | **(-) 02.16** | **66** | **0.1702** | **2** |
|  | **0.125** | **21.71 ± 0.75** | **(-) 04.40** | **69** | **0.5641** | **2** |
|  |  |  |  |  |  |  |
| **¶ The worms were maintained at 15˚C. Following hypochlorite treatment worms were grown at 25˚C on different RNAi feed till young adult stage. YA worms were transferred to Fudr overlaid plates and maintained at 20˚C for lifespan analysis.** | | | | | | |
