# Supplementary material for: A chromatin modifier integrates insulin/IGF‐1 signalling and dietary restriction to regulate longevity
Source: Aging Cell. 2016 Apr 2;15(4):694–705. doi: 10.1111/acel.12477 (PMC4933660; doi:10.1111/acel.12477)
Supplement: Supplementary file 3 — Table S2 List of primers used in the study. [file ACEL-15-694-s003.docx]

**Table S2: List of primers used in the study**

| **Cloning and Real time primers** | | | |
| --- | --- | --- | --- |
| **Gene name** | **Primer Name** | **Primer Sequence** | **Restriction site** |
| **RNAi primers** | | |  |
| ***zfp-1 2ac (cDNA)*** | **Forward Primer** | **GCTCTAGAGGA ACT AGT AGC AAT GAT GGA G** | **XbaI** |
|  | **Reverse Primer** | **CCGCTCGAGGCA ACG TTG GCA TCA AGT GGC AT** | **XhoI** |
| ***gfl-1(genomic)*** | **Forward Primer** | **TGTTTTTAACTGAATCCATCGCT** |  |
|  | **Reverse Primer** | **TCTCTGAAATGTGCTGAGTGTGT** |  |
| **For promoter Transgenic** | | |  |
| ***zfp-1 2a promoter (1kb)*** | **Forward Primer** | **acagtcgacGAT TGT GAT GGT GGT TTG GTG A** | **SalI** |
|  | **Reverse Primer** | **cctctagaTTT TCA GCA ATT TCG GGG GAC T** | **XbaI** |
| ***zfp-1 2c promoter (2.4kb)*** | **Forward Primer** | **cgcaagcttGTT GTT TCG TCT GCG CTT CTT** | **HindIII** |
|  | **Reverse Primer** | **acacctgcaggATG CAA CCT GGG TGC CGG** | **SbfI** |
| ***gfl-1 promoter (0.5 kb)*** | **Forward Primer** | **cgcaagcttGGT GTC TAC GTT TAA ACG CA** | **HindIII** |
|  | **Reverse Primer** | gcGGATCCGACTAGTTGTGCTGAAAAATAAC | **BamHI** |
| **DAF-16 ChIP qRT-PCR primers for *zfp-1* and *gfl-1* gene promoters** | | | |
| ***zfp-1 Peak 1*** | **Forward Primer** | **TTCGATTCCACCCACCACAG** |  |
|  | **Reverse Primer** | **GGCGAGAAGAGGAGGATGTG** |  |
| ***zfp-1 Peak 2*** | **Forward Primer** | **CACTAGGGTCTGCTGCATCC** |  |
|  | **Reverse Primer** | **TGCATACATACACGCGACGA** |  |
| ***zfp-1 Peak 3*** | **Forward Primer** | **CGTGATGTCGGTCTCTCCTAC** |  |
|  | **Reverse Primer** | **GTCACCAAACCACCATCACA** |  |
| ***zfp-1 Peak 4*** | **Forward Primer** | **CATTGTCGAAGGAGGTCCGC** |  |
|  | **Reverse Primer** | **GCCCAACCCATTTCATTTCGT** |  |
| ***zfp-1 Peak 5*** | **Forward Primer** | **TCCTCGCTTTGTCCTGCTTT** |  |
|  | **Reverse Primer** | **TTGCCTCACTATGTGCGTGT** |  |
| ***zfp-1 Peak 6*** | **Forward Primer** | **AATGATCGGTGTCCTCTGCC** |  |
|  | **Reverse Primer** | **CGCGTAGAGACACGTACACA** |  |
| ***zfp-1 distal*** | **Forward Primer** | **GGCATGTATCAAACCGCACC** |  |
|  | **Reverse Primer** | **CGCATACAGATCTCACCCCG** |  |
| ***gfl-1 peak 1*** | **Forward Primer** | **AACGACACTCCGTAATGCCA** |  |
|  | **Reverse Primer** | **AGCGATGGATTCAGTTAAAAACAC** |  |
| ***gfl-1 distal*** | **Forward Primer** | **GAGTTGAGTTGCGAAAGAATCT** |  |
|  | **Reverse Primer** | **TATTGAGGGTGTCGAATGACG** |  |
| **Real time primers for *zfp-1* and *gfl-1*** | | | |
| ***actin*** | **Forward Primer** | **CTCTTGCCCCATCAACCATG** |  |
|  | **Reverse Primer** | **CTTGCTTGGAGATCCACATC** |  |
| ***zfp-1 2a*** | **Forward Primer** | **GACGTGCAAGAGGAGCTTTC** |  |
|  | **Reverse Primer** | **GGATCGTTTATCGCTTTCTTCA** |  |
| ***zfp-1 2ac*** | **Forward Primer** | **TTCAGAATCACAGAGCAACACC** |  |
|  | **Reverse Primer** | **CATCAAGTGGCATGCGATAC** |  |
| ***gfl-l*** | **Forward Primer** | **GGA CAG TTT TCC TAA AGC CAT AC** |  |
|  | **Reverse Primer** | **GTT TTT CGA CAA CTC TGT ACG GA** |  |
| **Real time primers for DAF-16 target genes** | | | |
| ***sod-3*** | **Forward Primer** | **GGCTGTTTCGAAAGGGAATCTA** |  |
|  | **Reverse Primer** | **TCAGCTCCTTTGAAGGTTCTC** |  |
| ***mtl-1*** | **Forward Primer** | **AGTGTGACTGCAAAAACAAGCAA** |  |
|  | **Reverse Primer** | **TCCACTGCATTCACATTTGTCTC** |  |
| ***lys-7*** | **Forward Primer** | **GCCGTCAAACTTGGCATCTT** |  |
|  | **Reverse Primer** | **GGGTTGTATGCACGAACGAA** |  |
| ***zk742.4*** | **Forward Primer** | **GTGAGCCAGATTTGCCTCGT** |  |
|  | **Reverse Primer** | **TTATCGATCGTGCAGCCATTG** |  |
| ***hsp-12.6*** | **Forward Primer** | **TGGAGTTGTCAATGTCCTCG** |  |
|  | **Reverse Primer** | **GACTTCAATCTCTTTTGGGAGG** |  |
| ***sod-5*** | **Forward Primer** | **ATGGAGGAAGAGATTCCGTG** |  |
|  | **Reverse Primer** | **CAGTGTTCGCTCCGAAGAG** |  |
| ***scl-1*** | **Forward Primer** | **CAATCAAGCATTGTGGATGC** |  |
|  | **Reverse Primer** | **GGAATCCACGACCATTTTCC** |  |
| ***sip-1*** | **Forward Primer** | **AAGAGATCGTTCACTCGCCAG** |  |
|  | **Reverse Primer** | **AGCCAAGTCGACGTCCTTTG** |  |
| **DAF-16 ChIP qRT-PCR primers for DAF-16 target gene promoters** | | | |
| ***Psod-3*** | **Forward Primer** | **CCGAAAATTGACCTTTGAC** |  |
|  | **Reverse Primer** | **CAAAGACCTCATCAACAGCA** |  |
| ***Pmtl-1*** | **Forward Primer** | **GACAGGGCCACCCTCTTTTA** |  |
|  | **Reverse Primer** | **GCCTTCTTCTTTTTCTCACTGCC** |  |
| ***Phsp-12.6*** | **Forward Primer** | **ACAATATGGGCGGAGTCTGG** |  |
|  | **Reverse Primer** | **TCGCGCACAGATTCATGTCT** |  |
| ***Pclec-85*** | **Forward Primer** | **ACCACACCCAACAACCTCAG** |  |
|  | **Reverse Primer** | **CCGTGTGCATTGACCAAAGA** |  |
| ***Pdao-6*** | **Forward Primer** | **CAAGGCTCACGAGCTTTATGC** |  |
|  | **Reverse Primer** | **CAGACGGTCAAATGACACTGC** |  |
| ***Pzfp-1*** | **Forward Primer** | **CATTGTCGAAGGAGGTCCGC** |  |
|  | **Reverse Primer** | **GCCCAACCCATTTCATTTCGT** |  |
| ***Pgfl-l*** | **Forward Primer** | **AACGACACTCCGTAATGCCA** |  |
|  | **Reverse Primer** | **AGCGATGGATTCAGTTAAAAACAC** |  |
| ***Psod-3 distal*** | **Forward Primer** | **AATCGGGAACTTCACTCGGT** |  |
|  | **Reverse Primer** | **CTCGATCTGATACGTTTGTCCA** |  |
| **Real time primers for PHA-4 target genes** | | | |
| ***sod-1*** | **Forward Primer** | **AGGTCTCCAACGCGATTTTT** |  |
|  | **Reverse Primer** | **CCTGGTCATTTTCGGACTTC** |  |
| ***sod-2*** | **Forward Primer** | **CAACCGATCACAGGAGTCG** |  |
|  | **Reverse Primer** | **TTACAGGCTCCAAATCAGCA** |  |
| ***sod-4*** | **Forward Primer** | **ACGCGGTACTTCAGACCAAT** |  |
|  | **Reverse Primer** | **GAAGGGATGCTGTCGTTGTT** |  |
| ***cyp-32B1*** | **Forward Primer** | **GGTGTGTTGAAGTTATGGTTGGGACC** |  |
|  | **Reverse Primer** | **TGTCGCCGGTGCTGATTAAAAGAC** |  |
| ***cyp-33C8*** | **Forward Primer** | **CGCTGGATGATGTGCTCAACTACTGG** |  |
|  | **Reverse Primer** | **GCTTCTTCTGCTCTTTCAGGTAGG** |  |
| ***cyp-34A4*** | **Forward Primer** | **GATTTGAACAGGGTGACCCAGAAT** |  |
|  | **Reverse Primer** | **TCGATGACATGCTCACCACT** |  |
| ***cyp-37B1*** | **Forward Primer** | **GCTTGGAACGGGACTATTGAC** |  |
|  | **Reverse Primer** | **TTGTTCGAGGAAAACCTTGGCCTG** |  |
| ***ugt-16*** | **Forward Primer** | **CTTGCTGACGATCGACTAACC** |  |
|  | **Reverse Primer** | **CGGTCTGTATGGCTTCTCTAAG** |  |
| **PHA-4 ChIP qRT-PCR primers for PHA-4 target gene promoters** | | | |
| ***zfp-1 Peak 1*** | **Forward Primer** | **TTCGATTCCACCCACCACAG** |  |
|  | **Reverse Primer** | **GGCGAGAAGAGGAGGATGTG** |  |
| ***zfp-1 Peak 2*** | **Forward Primer** | **CACTAGGGTCTGCTGCATCC** |  |
|  | **Reverse Primer** | **TGCATACATACACGCGACGA** |  |
| ***zfp-1 Peak 4*** | **Forward Primer** | **CATTGTCGAAGGAGGTCCGC** |  |
|  | **Reverse Primer** | **GCCCAACCCATTTCATTTCGT** |  |
| ***zfp-1 distal*** | **Forward Primer** | **GAAGACCAGACTCATCAATTTG** |  |
|  | **Reverse Primer** | **CTCCAACAATACGGATATCCAC** |  |
| ***gfl-1 peak 1*** | **Forward Primer** | **AACGACACTCCGTAATGCCA** |  |
|  | **Reverse Primer** | **AGCGATGGATTCAGTTAAAAACAC** |  |
| ***gfl-1 distal*** | **Forward Primer** | **CCGCGATAACTCCTCTCGAA** |  |
|  | **Reverse Primer** | **GCCAAAATGCCTACTTGTGTGC** |  |
| **RNAi efficiency check primers** | | | |
| ***zfp-1 (2a)*** | **Forward Primer** | **GACGTGCAAGAGGAGCTTTC** |  |
|  | **Reverse Primer** | **GGATCGTTTATCGCTTTCTTCA** |  |
| ***zfp-1 (2ac)*** | **Forward Primer** | **CGCTGCACAATCTCAACCAT** |  |
|  | **Reverse Primer** | **ACGCTGGCGCGTTTTGAGTA** |  |
| ***gfl-l*** | **Forward Primer** | **GTCTGAAAAATTGTGAAAAAAGCG** |  |
|  | **Reverse Primer** | **CGGTAAACAATGCGCACGTG** |  |
