# Supplementary material for: A chromatin modifier integrates insulin/IGF‐1 signalling and dietary restriction to regulate longevity
Source: Aging Cell. 2016 Apr 2;15(4):694–705. doi: 10.1111/acel.12477 (PMC4933660; doi:10.1111/acel.12477)
Supplement: Supplementary file 4 — Table S3 Details of life span experiments performed (Individual experiments used for consolidation). [file ACEL-15-694-s004.docx]

**Table S3: Details of life span experiments performed (Individual experiments used for consolidation)**

| **Genetic Background** | **RNAi** | **Mean ± SEM** | **Percentage increase(+) or decrease (-) w.r.t. control** | **n (Number of animals)** | ***P-*value** |
| --- | --- | --- | --- | --- | --- |
| **Wild-type (Set 1)** | **control** | **18.44 ± 0.25** |  | **82** |  |
|  | ***daf-16*** | **14.20 ± 0.16** | **(-) 22.99** | **66** | **≤0.0001** |
|  | ***zfp-1(2ac)*** | **18.03 ± 0.18** | **(-) 02.22** | **77** | **0.0185** |
|  | ***gfl-1*** | **17.93 ± 0.16** | **(-) 02.77** | **84** | **0.0062** |
|  |  |  |  |  |  |
| ***daf-2(e1370)* (Set 1)** | **control** | **41.13 ± 0.88** |  | **86** |  |
|  | ***daf-16*** | **18.17 ± 0.17** | **(-) 55.82** | **60** | **≤0.0001** |
|  | ***zfp-1(2ac)*** | **30.15 ± 1.51** | **(-) 26.70** | **33** | **≤0.0001** |
|  | ***gfl-1*** | **41.56 ± 0.87** | **(+) 01.04** | **80** | **0.8857** |
|  |  |  |  |  |  |
| ***daf-16(mgDf50)* (Set 1)** | **control** | **17.81 ± 0.26** |  | **72** |  |
|  | ***zfp-1(2ac)*** | **16.36 ± 0.36** | **(-) 08.14** | **46** | **0.0032** |
|  | ***gfl-1*** | **16.94 ± 0.35** | **(-) 04.88** | **31** | **0.0375** |
|  |  |  |  |  |  |
| ***daf-16(mgDf50);daf-2(e1370)* (Set 1)** | **control** | **16.55 ± 0.43** |  | **30** |  |
|  | ***zfp-1(2ac)*** | **15.67 ± 0.17** | **(-) 05.32** | **40** | **0.0090** |
|  | ***gfl-1*** | **16.86 ± 0.34** | **(+) 01.87** | **30** | **0.8665** |
|  |  |  |  |  |  |
| ***daf-16(mgDf50);daf-2(e1370);daf-16(a)* (Set 1)** | **control** | **34.91 ± 0.88** |  | **69** |  |
|  | ***daf-16*** | **15.29 ± 0.24** | **(-) 56.20** | **55** | **≤0.0001** |
|  | ***zfp-1(2ac)*** | **30.70 ± 0.76** | **(-) 12.06** | **66** | **≤0.0001** |
|  | ***gfl-1*** | **28.89 ± 0.84** | **(-) 17.24** | **71** | **≤0.0001** |
|  |  |  |  |  |  |
| ***daf-16(mgDf50);daf-2(e1370);daf-16(f)* (Set 1)** | **control** | **50.70 ± 2.26** |  | **30** |  |
|  | ***daf-16*** | **16.80 ± 0.27** | **(-) 66.86** | **35** | **≤0.0001** |
|  | ***zfp-1(2ac)*** | **33.16 ± 1.51** | **(-) 34.60** | **44** | **≤0.0001** |
|  | ***gfl-1*** | **48.89 ± 1.64** | **(-) 03.57** | **53** | **0.3077** |
|  |  |  |  |  |  |
| **Wild-type (Set 2)** | **control** | **20.29 ± 0.45** |  | **75** |  |
|  | ***daf-16*** | **15.49 ± 0.41** | **(-) 23.67** | **43** | **≤0.0001** |
|  | ***zfp-1(2ac)*** | **20.57 ± 0.46** | **(+) 01.38** | **73** | **0.6502** |
|  | ***gfl-1*** | **19.46 ± 0.50** | **(-) 04.09** | **81** | **0.3673** |
|  |  |  |  |  |  |
| ***daf-2(e1370)* (Set 2)** | **control** | **43.75 ± 1.47** |  | **61** |  |
|  | ***daf-16*** | **17.97 ± 0.27** | **(-) 58.92** | **60** | **≤0.0001** |
|  | ***zfp-1(2ac)*** | **27.27 ± 1.15** | **(-) 37.67** | **44** | **≤0.0001** |
|  | ***gfl-1*** | **40.27 ± 1.10** | **(-) 07.95** | **92** | **0.0075** |
|  |  |  |  |  |  |
| ***daf-16(mgDf50)* (Set 2)** | **control** | **18.57 ± 0.23** |  | **84** |  |
|  | ***zfp-1(2ac)*** | **16.52 ± 0.28** | **(-) 11.04** | **61** | **≤0.0001** |
|  | ***gfl-1*** | **17.13 ± 0.26** | **(-) 07.55** | **90** | **0.0003** |
|  |  |  |  |  |  |
| ***daf-16(mgDf50);daf-2(e1370)***  **(Set-2)** | **control** | **20.26 ± 0.29** |  | **111** |  |
|  | ***zfp-1(2ac)*** | **20.02 ± 0.44** | **(-) 01.18** | **57** | **0.7644** |
|  | **control** | **21.29 ± 0.44** |  | **58** |  |
|  | ***gfl-1*** | **19.69 ± 0.38** | **(-) 07.51** | **65** | **0.0018** |
|  |  |  |  |  |  |
| ***daf-16(mgDf50);daf-2(e1370);daf-16(a)* (Set 2)** | **control** | **30.78 ± 0.49** |  | **64** |  |
|  | ***daf-16*** | **19.46 ± 0.36** | **(-) 36.77** | **57** | **≤0.0001** |
|  | ***zfp-1(2ac)*** | **29.80 ± 0.35** | **(-) 03.18** | **69** | **0.0181** |
|  | ***gfl-1*** | **26.87 ± 0.44** | **(-) 12.70** | **61** | **≤0.0001** |
|  |  |  |  |  |  |
| ***daf-16(mgDf50);daf-2(e1370);daf-16(f)* (Set 2)** | **control** | **58.10 ± 1.32** |  | **58** |  |
|  | ***daf-16*** | **17.40 ± 0.46** | **(-) 70.05** | **35** | **≤0.0001** |
|  | ***zfp-1(2ac)*** | **44.23 ± 1.17** | **(-) 23.87** | **65** | **≤0.0001** |
|  | ***gfl-1*** | **56.47 ± 1.24** | **(-) 02.80** | **62** | **0.1443** |
|  |  |  |  |  |  |
| **Wild-type (Set 3)** | **control** | **20.45 ± 0.39** |  | **62** |  |
|  | ***daf-16*** | **16.00 ± 0.00** | **(-) 21.76** | **56** | **≤0.0001** |
|  | ***zfp-1(2ac)*** | **20.49 ± 0.37** | **(+) 00.20** | **37** | **0.7800** |
|  | ***gfl-1*** | **20.37 ± 0.28** | **(-) 00.40** | **62** | **0.4687** |
|  |  |  |  |  |  |
| ***daf-2(e1370)* (Set 3)** | **control** | **43.53 ± 0.78** |  | **91** |  |
|  | ***daf-16*** | **18.38 ± 0.26** | **(-) 57.78** | **72** | **≤0.0001** |
|  | ***zfp-1(2ac)*** | **31.11 ± 1.25** | **(-) 28.53** | **53** | **≤0.0001** |
|  | ***gfl-1*** | **38.11 ± 0.79** | **(-) 12.45** | **89** | **≤0.0001** |
|  |  |  |  |  |  |
| ***daf-16(mgDf50);daf-2(e1370)* (Set 3)** | **control** | **18.00 ± 0.30** |  | **77** |  |
|  | ***zfp-1(2ac)*** | **16.71 ± 0.38** | **(-) 07.20** | **56** | **0.0142** |
|  | ***gfl-1*** | **15.15 ± 0.30** | **(-) 15.83** | **62** | **≤0.0001** |
|  |  |  |  |  |  |
| ***daf-16(mgDf50);daf-2(e1370);daf-16(a)* (Set 3)** | **control** | **31.50 ± 0.35** |  | **64** |  |
|  | ***daf-16*** | **23.30 ± 0.65** | **(-) 26.03** | **53** | **≤0.0001** |
|  | ***zfp-1(2ac)*** | **30.50 ± 0.29** | **(-) 03.17** | **64** | **0.0104** |
|  | ***gfl-1*** | **27.67 ± 0.34** | **(-) 12.15** | **55** | **≤0.0001** |
|  |  |  |  |  |  |
| ***daf-16(mgDf50);daf-2(e1370);daf-16(f)* (Set 3)** | **control** | **57.43 ± 0.99** |  | **72** |  |
|  | ***daf-16*** | **18.98 ± 0.41** | **(-) 66.95** | **48** | **≤0.0001** |
|  | ***zfp-1(2ac)*** | **46.52 ± 1.22** | **(-) 18.99** | **63** | **≤0.0001** |
|  | ***gfl-1*** | **56.74 ± 1.31** | **(-) 01.20** | **35** | **0.2511** |
|  |  |  |  |  |  |
| **Wild-type (Set 4)** | **control** | **18.81 ± 0.45** |  | **43** |  |
|  | ***daf-16*** | **15.00 ± 0.20** | **(-) 20.25** | **31** | **≤0.0001** |
|  | ***zfp-1(2ac)*** | **19.86 ± 0.74** | **(+) 05.58** | **30** | **0.0971** |
|  | **control** | **18.63 ± 0.22** |  | **113** |  |
|  | ***gfl-1*** | **18.30 ± 0.33** | **(-) 01.77** | **66** | **0.6268** |
|  |  |  |  |  |  |
| ***daf-2(e1370)* (Set 4)** | **control** | **49.42 ± 1.01** |  | **73** |  |
|  | ***daf-16*** | **17.42 ± 0.31** | **(-) 64.75** | **56** | **≤0.0001** |
|  | ***zfp-1(2ac)*** | **31.04 ± 2.02** | **(-) 37.20** | **30** | **≤0.0001** |
|  | ***gfl-1*** | **44.11 ± 1.15** | **(-) 10.74** | **47** | **≤0.0001** |
|  |  |  |  |  |  |
| ***daf-16(mgDf50);daf-2(e1370)* (Set 4)** | **control** | **17.11 ± 0.31** |  | **76** |  |
|  | ***zfp-1(2ac)*** | **16.77 ± 0.29** | **(-) 01.98** | **71** | **0.4606** |
|  | ***gfl-1*** | **16.37 ± 0.27** | **(-) 04.32** | **87** | **0.0591** |
|  |  |  |  |  |  |
| ***daf-16(mgDf50);daf-2(e1370);daf-16(a)* (Set 4)** | **control** | **33.28 ± 0.57** |  | **83** |  |
|  | ***daf-16*** | **25.31 ± 0.39** | **(-) 23.94** | **78** | **≤0.0001** |
|  | ***zfp-1(2ac)*** | **34.01 ± 0.58** | **(+) 02.19** | **114** | **0.1597** |
|  | ***gfl-1*** | **31.24 ± 0.71** | **(-) 06.13** | **86** | **0.0681** |
|  |  |  |  |  |  |
| ***daf-16(mgDf50);daf-2(e1370);daf-16(f)* (Set 4)** | **control** | **59.35 ± 0.98** |  | **89** |  |
|  | ***daf-16*** | **21.65 ± 0.41** | **(-) 63.52** | **96** | **≤0.0001** |
|  | ***zfp-1(2ac)*** | **45.22 ± 0.91** | **(-) 23.80** | **139** | **≤0.0001** |
|  | ***gfl-1*** | **52.39 ± 1.05** | **(-) 11.72** | **114** | **≤0.0001** |
|  |  |  |  |  |  |
| **Wild-type (Set 5)** | **control** | **21.45 ± 0.53** |  | **61** |  |
|  | ***daf-16*** | **14.50 ± 0.13** | **(-) 32.40** | **72** | **≤0.0001** |
|  | ***zfp-1(2ac)*** | **19.22 ± 0.30** | **(-) 10.40** | **46** | **0.0005** |
|  | **control** | **20.34 ± 0.36** |  | **76** |  |
|  | ***gfl-1*** | **18.79 ± 0.39** | **(-) 07.62** | **52** | **0.0050** |
|  |  |  |  |  |  |
| ***daf-16(mgDf50)* (Set 3)** | **control** | **15.17 ± 0.26** |  | **83** |  |
|  | ***zfp-1(2ac)*** | **14.23 ± 0.27** | **(-) 06.20** | **56** | **0.0109** |
|  | ***gfl-1*** | **14.09 ± 0.20** | **(-) 07.12** | **77** | **0.0011** |
|  |  |  |  |  |  |
| **Wild-type (Set 6)** | **control** | **19.89 ± 0.31** |  | **62** |  |
|  | ***daf-16*** | **15.74 ± 0.31** | **(-) 20.86** | **42** | **≤0.0001** |
|  | ***zfp-1(2ac)*** | **20.79 ± 0.28** | **(+) 04.52** | **66** | **0.0508** |
|  | **control** | **18.38 ± 0.30** |  | **82** |  |
|  | ***gfl-1*** | **17.55 ± 0.38** | **(-) 04.52** | **67** | **0.2064** |
|  |  |  |  |  |  |
| ***daf-16(mgDf50)* (4)** | **control** | **15.63 ± 0.19** |  | **61** |  |
|  | ***zfp-1(2ac)*** | **17.76 ± 0.35** | **(+) 13.63** | **45** | **≤0.0001** |
|  | ***gfl-1*** | **16.55 ± 0.17** | **(+) 05.89** | **87** | **0.0007** |
|  |  |  |  |  |  |
| **Wild-type (Set 7)** | **control** | **22.82 ± 0.49** |  | **101** |  |
|  | ***daf-16*** | **14.83 ± 0.14** | **(-) 35.01** | **83** | **≤0.0001** |
|  | ***zfp-1(2ac)*** | **20.94 ± 0.43** | **(-) 08.23** | **49** | **0.0015** |
|  |  |  |  |  |  |
| **Wild-type (for *let-363*) (Set 1)** | **control** | **17.76 ± 0.36** |  | **93** |  |
|  | ***daf-16*** | **14.41 ± 0.29** | **(-) 18.86** | **80** | **≤0.0001** |
|  | ***zfp-1(2ac)*** | **16.48 ± 0.24** | **(-) 07.20** | **71** | **0.0005** |
|  | ***gfl-1*** | **17.02 ± 0.28** | **(-) 04.17** | **90** | **0.0070** |
|  |  |  |  |  |  |
| ***let-363 (ok3018)*(Set 1)** | **control** | **29.15 ± 0.45** |  | **76** |  |
|  | ***daf-16*** | **20.15 ± 0.40** | **(-) 30.87** | **65** | **≤0.0001** |
|  | ***zfp-1(2ac)*** | **19.20 ± 0.33** | **(-) 34.13** | **53** | **≤0.0001** |
|  | ***gfl-1*** | **24.04 ± 0.47** | **(-) 17.53** | **71** | **≤0.0001** |
|  |  |  |  |  |  |
| **Wild-type (for *let-363*) (Set 2)** | **control** | **16.56 ± 0.31** |  | **78** |  |
|  | ***daf-16*** | **14.18 ± 0.24** | **(-) 14.37** | **80** | **≤0.0001** |
|  | ***zfp-1(2ac)*** | **15.01 ± 0.22** | **(-) 09.36** | **72** | **≤0.0001** |
|  | ***gfl-1*** | **15.60 ± 0.27** | **(-) 05.79** | **75** | **0.0164** |
|  |  |  |  |  |  |
| ***let-363 (ok3018)*(Set 2)** | **control** | **30.22 ± 0.81** |  | **40** |  |
|  | ***daf-16*** | **20.48 ± 0.33** | **(-) 32.23** | **52** | **≤0.0001** |
|  | ***zfp-1(2ac)*** | **21.26 ± 0.66** | **(-) 29.65** | **35** | **≤0.0001** |
|  | ***gfl-1*** | **21.32 ± 0.54** | **(-) 29.45** | **44** | **≤0.0001** |
|  |  |  |  |  |  |
| **Wild-type (for *let-363*) (Set 3)** | **control** | **16.46 ± 0.27** |  | **76** |  |
|  | ***daf-16*** | **13.91 ± 0.24** | **(-) 15.49** | **80** | **≤0.0001** |
|  | ***zfp-1(2ac)*** | **16.55 ± 0.43** | **(+) 00.55** | **40** | **0.7113** |
|  | ***gfl-1*** | **15.51 ± 0.25** | **(-) 05.77** | **77** | **0.0122** |
|  |  |  |  |  |  |
| ***let-363 (ok3018)*(Set 3)** | **control** | **28.73 ± 0.58** |  | **84** |  |
|  | ***daf-16*** | **19.44 ± 0.37** | **(-) 32.33** | **55** | **≤0.0001** |
|  | ***zfp-1(2ac)*** | **25.79 ± 0.45** | **(-) 10.23** | **85** | **≤0.0001** |
|  | ***gfl-1*** | **24.83 ± 0.44** | **(-) 13.57** | **87** | **≤0.0001** |
|  |  |  |  |  |  |
| **Wild-type (for *glp-1*)^¶^ (Set 1)** | **control** | **18.85 ± 0.33** |  | **110** |  |
|  | ***daf-16*** | **14.91 ± 0.29** | **(-) 20.90** | **80** | **≤0.0001** |
|  | ***zfp-1(2ac)*** | **17.72 ± 0.19** | **(-) 05.99** | **123** | **≤0.0001** |
|  | ***gfl-1*** | **16.60 ± 0.29** | **(-) 11.93** | **114** | **≤0.0001** |
|  |  |  |  |  |  |
| ***glp-1 (e2141)* ^¶^ (Set 1)** | **control** | **25.06 ± 0.66** |  | **93** |  |
|  | ***daf-16*** | **15.95 ± 0.33** | **(-) 36.35** | **60** | **≤0.0001** |
|  | ***zfp-1(2ac)*** | **18.53 ± 0.40** | **(-) 26.06** | **80** | **≤0.0001** |
|  | ***gfl-1*** | **19.36 ± 0.57** | **(-) 22.74** | **76** | **≤0.0001** |
|  |  |  |  |  |  |
| **Wild-type (for *glp-1*)^¶^ (Set 2)** | **control** | **17.67 ± 0.41** |  | **73** |  |
|  | ***daf-16*** | **14.36 ± 0.25** | **(-) 18.73** | **81** | **≤0.0001** |
|  | ***zfp-1(2ac)*** | **16.14 ± 0.29** | **(-) 08.66** | **81** | **0.0020** |
|  | ***gfl-1*** | **15.53 ± 0.29** | **(-) 12.11** | **89** | **≤0.0001** |
|  |  |  |  |  |  |
| ***glp-1 (e2141)* ^¶^ (Set 2)** | **control** | **23.25 ± 0.70** |  | **87** |  |
|  | ***daf-16*** | **14.63 ± 0.31** | **(-) 37.07** | **68** | **≤0.0001** |
|  | ***zfp-1(2ac)*** | **19.98 ± 0.82** | **(-) 14.06** | **44** | **0.0041** |
|  | ***gfl-1*** | **19.78 ± 0.41** | **(-) 14.92** | **79** | **≤0.0001** |
|  |  |  |  |  |  |
| **Wild-type (for *eat* mutants) (Set 1)** | **control** | **19.89 ± 0.31** |  | **62** |  |
|  | ***pha-4*** | **15.28 ± 0.35** | **(-) 23.17** | **74** | **≤0.0001** |
|  | ***zfp-1(2ac)*** | **20.79 ± 0.28** | **(+) 04.52** | **66** | **0.0508** |
|  | ***gfl-1*** | **19.32 ± 0.25** | **(-) 02.86** | **62** | **0.1558** |
|  |  |  |  |  |  |
| ***eat-2(ad1116)* (Set 1)** | **control** | **34.64 ± 0.65** |  | **72** |  |
|  | ***pha-4*** | **19.59 ± 0.60** | **(-) 43.44** | **61** | **≤0.0001** |
|  | ***zfp-1(2ac)*** | **26.84 ± 0.48** | **(-) 22.52** | **62** | **≤0.0001** |
|  | ***gfl-1*** | **26.58 ± 0.38** | **(-) 23.26** | **40** | **≤0.0001** |
|  |  |  |  |  |  |
| ***eat-2(ad465)* (Set 1)** | **control** | **29.04 ± 0.48** |  | **72** |  |
|  | ***pha-4*** | **19.21 ± 0.39** | **(-) 33.84** | **68** | **≤0.0001** |
|  | ***zfp-1(2ac)*** | **23.84 ± 0.32** | **(-) 17.90** | **70** | **≤0.0001** |
|  | ***gfl-1*** | **25.69 ± 0.26** | **(-) 11.53** | **85** | **≤0.0001** |
|  |  |  |  |  |  |
| ***eat-2(ad1113)* (Set 1)** | **control** | **23.50 ± 0.46** |  | **54** |  |
|  | ***pha-4*** | **15.37 ± 0.28** | **(-) 34.59** | **41** | **≤0.0001** |
|  | ***zfp-1(2ac)*** | **21.08 ± 0.42** | **(-) 10.29** | **71** | **0.0008** |
|  | ***gfl-1*** | **19.19 ± 0.34** | **(-) 18.34** | **64** | **≤0.0001** |
|  |  |  |  |  |  |
| **Wild-type (for *eat* mutants) (Set 2)** | **control** | **20.29 ± 0.45** |  | **52** |  |
|  | ***pha-4*** | **15.58 ± 0.41** | **(-) 23.21** | **50** | **≤0.0001** |
|  | ***zfp-1(2ac)*** | **20.57 ± 0.46** | **(+) 01.38** | **55** | **0.6502** |
|  | ***gfl-1*** | **19.46 ± 0.50** | **(-) 04.09** | **68** | **0.3673** |
|  | **control** | **22.82 ± 0.49** |  | **101** |  |
|  | ***zfp-1(2ac)*** | **20.94 ± 0.43** | **(-) 08.23** | **49** | **0.0015** |
|  |  |  |  |  |  |
| ***eat-2(ad1116)* (Set 2)** | **control** | **32.65 ± 0.84** |  | **81** |  |
|  | ***pha-4*** | **20.13 ± 0.57** | **(-) 38.35** | **39** | **≤0.0001** |
|  | ***zfp-1(2ac)*** | **27.30 ± 0.50** | **(-) 16.38** | **80** | **≤0.0001** |
|  | ***gfl-1*** | **21.80 ± 0.40** | **(-) 33.23** | **60** | **≤0.0001** |
|  |  |  |  |  |  |
| ***eat-2(ad465)* (Set 2)** | **control** | **34.56 ± 1.12** |  | **66** |  |
|  | ***pha-4*** | **20.42 ± 0.73** | **(-) 40.91** | **57** | **≤0.0001** |
|  | ***zfp-1(2ac)*** | **30.53 ± 0.57** | **(-) 11.56** | **72** | **≤0.0001** |
|  | ***gfl-1*** | **29.54 ± 0.65** | **(-) 14.52** | **62** | **≤0.0001** |
|  |  |  |  |  |  |
| ***eat-2(ad1113)* (Set 2)** | **control** | **24.09 ± 0.70** |  | **55** |  |
|  | ***pha-4*** | **14.98 ± 0.44** | **(-) 37.81** | **47** | **≤0.0001** |
|  | ***gfl-1*** | **15.71 ± 0.29** | **(-) 34.79** | **68** | **≤0.0001** |
|  |  |  |  |  |  |
| **Wild-type (for *eat* mutants) (Set3)** | **control** | **19.51 ± 0.59** |  | **75** |  |
|  | ***pha-4*** | **15.47 ± 0.38** | **(-) 20.70** | **51** | **≤0.0001** |
|  | ***zfp-1(2ac)*** | **18.05 ± 0.46** | **(-) 07.48** | **88** | **0.0432** |
|  | ***gfl-1*** | **17.21 ± 0.47** | **(-) 11.79** | **62** | **0.0030** |
|  |  |  |  |  |  |
| ***eat-2(ad1116)* (Set 3)** | **control** | **35.96 ± 0.78** |  | **47** |  |
|  | ***pha-4*** | **20.21 ± 0.80** | **(-) 43.79** | **58** | **≤0.0001** |
|  | ***zfp-1(2ac)*** | **28.89 ± 0.63** | **(-) 19.66** | **54** | **≤0.0001** |
|  | ***gfl-1*** | **29.06 ± 0.66** | **(-) 19.19** | **79** | **≤0.0001** |
|  |  |  |  |  |  |
| ***eat-2(ad465)* (Set 3)** | **control** | **31.53 ± 0.83** |  | **104** |  |
|  | ***pha-4*** | **23.36 ± 1.23** | **(-) 25.91** | **42** | **≤0.0001** |
|  | ***zfp-1(2ac)*** | **28.26 ± 0.40** | **(-) 10.37** | **92** | **≤0.0001** |
|  | ***gfl-1*** | **29.59 ± 0.50** | **(-) 06.15** | **64** | **0.0053** |
|  |  |  |  |  |  |
| ***eat-2(ad1113)*( Set 3)** | **control** | **23.07 ± 0.72** |  | **73** |  |
|  | ***pha-4*** | **15.71 ± 0.64** | **(-) 31.90** | **42** | **≤0.0001** |
|  | ***zfp-1(2ac)*** | **18.84 ± 0.59** | **(-) 18.33** | **50** | **≤0.0001** |
|  | ***gfl-1*** | **17.94 ± 0.55** | **(-) 22.23** | **53** | **≤0.0001** |
|  |  |  |  |  |  |
| ***rrf-3(pk1426)*** | **control** | **18.92 ± 0.50** |  | **53** |  |
|  | ***zfp-1(2ac)*** | **21.34 ± 0.44** | **(+) 12.79** | **74** | **0.0007** |
|  | ***gfl-1*** | **17.96 ± 0.46** | **(-) 05.07** | **52** | **0.1054** |
|  |  |  |  |  |  |
| ***eat-2(ad1116);rrf-3(pk1426)*** | **control** | **33.69 ± 0.85** |  | **64** |  |
|  | ***zfp-1(2ac)*** | **26.15 ± 0.53** | **(-) 22.38** | **62** | **≤0.0001** |
|  | ***gfl-1*** | **26.18 ± 0.67** | **(-) 22.29** | **56** | **≤0.0001** |
|  |  |  |  |  |  |
| **Wild-type (Set 1)** | **control** | **20.37 ± 0.26** |  | **87** |  |
|  | ***drl-1*** | **25.53 ± 0.56** | **(+) 25.33** | **45** | **≤0.0001** |
|  |  |  |  |  |  |
| ***zfp-1(ok554)* (Set 1)** | **control** | **20.07 ± 0.53** |  | **30** |  |
|  | ***drl-1*** | **22.28 ± 0.63** | **(+) 11.01** | **25** | **0.0037** |
|  |  |  |  |  |  |
| **Wild-type (Set 2)** | **control** | **18.44 ± 0.25** |  | **82** |  |
|  | ***drl-1*** | **22.38 ± 0.51** | **(+) 21.37** | **71** | **≤0.0001** |
|  |  |  |  |  |  |
| ***zfp-1(ok554)* (Set 2)** | **control** | **19.08 ± 0.44** |  | **49** |  |
|  | ***drl-1*** | **17.00 ± 0.50** | **(-) 10.90** | **35** | **0.0271** |
|  |  |  |  |  |  |
| **Wild-type (Set 1)** | **control** | **21.33 ± 0.42** |  | **79** |  |
|  | ***drl-1*** | **27.20 ± 0.43** | **(+) 27.52** | **105** | **≤0.0001** |
| ***gfl-1(gk321)* (Set 1)** | **control** | **19.83 ± 0.42** |  | **76** |  |
|  | ***drl-1*** | **26.54 ± 0.51** | **(+) 33.84** | **103** | **≤0.0001** |
|  |  |  |  |  |  |
| **Wild-type (Set 2)** | **control** | **20.53 ± 0.34** |  | **95** |  |
|  | ***drl-1*** | **26.59 ± 0.38** | **(+) 29.52** | **110** | **≤0.0001** |
|  |  |  |  |  |  |
| ***gfl-1(gk321)* (Set 2)** | **control** | **22.00 ± 0.49** |  | **60** |  |
|  | ***drl-1*** | **31.07 ± 0.38** | **(+) 41.23** | **105** | **≤0.0001** |
|  |  |  |  |  |  |
| **BDR Lifespan** | **OD of OP50** |  |  |  |  |
| **Wild-type (Set 1)** | **3.0** | **25.15 ± 0.60** |  | **46** |  |
|  | **1.0** | **33.16 ± 0.75** | **(+) 31.84** | **43** | **≤0.0001** |
|  | **0.5** | **35.95 ± 1.20** | **(+) 42.94** | **41** | **≤0.0001** |
|  | **0.25** | **41.33 ± 1.06** | **(+) 64.33** | **46** | **≤0.0001** |
|  | **0.125** | **31.95 ± 1.31** | **(+) 27.03** | **43** | **≤0.0001** |
|  |  |  |  |  |  |
| ***zfp-1(ok554)* (Set 1)** | **3.0** | **22.81 ± 1.03** |  | **31** |  |
|  | **1.0** | **25.00 ± 2.16** | **(+) 09.60** | **22** | **0.0643** |
|  | **0.5** | **23.27 ± 1.24** | **(+) 02.02** | **33** | **0.5765** |
|  | **0.25** | **22.21 ± 1.17** | **(-) 02.63** | **33** | **0.8366** |
|  | **0.125** | **21.44 ± 1.12** | **(-) 06.00** | **36** | **0.4948** |
|  |  |  |  |  |  |
| **Wild-type (Set 2)** | **3.0** | **24.46 ± 0.89** |  | **39** |  |
|  | **1.0** | **28.69 ± 1.02** | **(+) 17.29** | **42** | **0.0016** |
|  | **0.5** | **29.49 ± 1.08** | **(+) 20.56** | **43** | **≤0.0001** |
|  | **0.25** | **28.58 ± 1.18** | **(+) 16.84** | **36** | **0.0027** |
|  | **0.125** | **27.54 ± 1.15** | **(+) 12.59** | **46** | **0.0087** |
|  |  |  |  |  |  |
| ***zfp-1(ok554)* (Set 2)** | **3.0** | **22.62 ± 0.97** |  | **34** |  |
|  | **1.0** | **22.04 ± 1.19** | **(-) 02.56** | **26** | **0.7785** |
|  | **0.5** | **20.74 ± 1.18** | **(-) 08.31** | **35** | **0.4317** |
|  | **0.25** | **24.18 ± 1.53** | **(+) 06.89** | **33** | **0.1118** |
|  | **0.125** | **22.00 ± 0.99** | **(-) 02.74** | **33** | **0.6727** |
| **¶ The worms were maintained at 15˚C. Following hypochlorite treatment worms were grown at 25˚C on different RNAi feed till young adult stage. YA worms were transferred to Fudr overlaid plates and maintained at 20˚C for lifespan analysis.** | | | | | |
