# Supplementary material for: A chromatin modifier integrates insulin/IGF‐1 signalling and dietary restriction to regulate longevity
Source: Aging Cell. 2016 Apr 2;15(4):694–705. doi: 10.1111/acel.12477 (PMC4933660; doi:10.1111/acel.12477)
Supplement: Supplementary file 6 — Data S1 Materials and methods. [file ACEL-15-694-s006.docx]

**Materials and methods**

***Strains***

Unless otherwise mentioned, all strains were maintained at 20 ^o^C using standard *C. elegans* techniques (Stiernagle, 2006). Strains used in the study are: N2 Bristol as wild-type, *daf-2(e1370)III*, *daf-16(mgDf50)I*, *daf-16(mgDf50)I;daf-2(e1370)III*, *daf-16(mgDf50)I;daf-2(e1370)III;daf-16a*, *daf-16(mgDf50)I;daf-2(e1370)III;daf-16f*, *rrf-3 (pk1426)II*, *eat-2(ad1116)II*, *eat-2(ad1113)II*, *eat-2(ad465) II*, *eat-2(ad1116);rrf-3(pk1426)II*, *zfp-1(ok554)III* (outcrossed 2X), *gfl-1(gk321)IV*, *Psod-3::gfp (muIs84)*, *daf-2(e1370)III; Psod-3::gfp*, *daf-16(mu86); Psod-3::gfp*, *daf-16(mu86)I;daf-2(e1370)III; Psod-3::gfp*, *mtl-1::rfp (CF2222)*, *Phsp-16.2::gfp (CF2070)*, *Pgpd-2::gfp (CF2893)*, *Pdod-11::rfp (CF2124)*, *glp-1(e2141)*, *let-363(ok3018) I/hT2 [bli-4(e937) let-?(q782) qIs48](I;III)*, (*unc-119(ed3) III; wgIs37* (*OP37*), *zfp-1(ok554);OP37*.

***Generation of RNAi clones***

The *zfp-1(2ac)* (752 bp) was amplified from wild-type cDNA using the Taq DNA polymerase (NEB, USA). *XbaI* and *XhoI* sites were incorporated in the 5’ and 3’ primers, respectively. The amplified products were purified using a PCR Purification Kit (Qiagen, Germany) and digested using *XbaI* and *XhoI* restriction enzymes (NEB, USA). Following Phenol:Chloroform:Isoamylalcohol (Sigma, USA) extraction, the fragments were cloned into the *pL4440* vector (Addgene, USA). Primers used are listed in Table S2.

***Life span***

Life span analysis was performed as described previously (Chamoli et al., 2014). NGM RNAi plates were prepared by supplementing Nematode Growth Media (NGM) media with 100 µg/ml ampicillin and 2 mM IPTG. RNAi bacteria were grown overnight at 37°C in LB media containing 100 µg/ml ampicillin and 12.5 µg/ml tetracycline. The cultures were diluted next day (1:100 v/v) in LB containing 100 µg/ml ampicillin and grown at 37°C till an OD_600_ of 0.6 was attained. The bacterial pellets were then resuspended in (1:10 v/v) in 1X M9 buffer containing 1 mM IPTG and 100 µg/ml ampicillin.

Gravid adult worms were bleached and the eggs were hatched on plates containing the respective RNAi bacteria. When the worms reached gravid adult stage, they were transferred to respective RNAi plates overlaid with 5-fluorodeoxyuridine (FUDR; final concentration of 0.1 mg/ml) (Hosono et al., 1982). Worms were scored as dead or alive by tapping them with a platinum wire every 2-3 days. Life span graph was plotted with percentage alive on Y axis and the number of days on the X axis. Statistical analyses for survival were conducted using Mantel-Cox log rank test through OASIS software available at http://sbi.postech.ac.kr/oasis (Yang et al., 2011). Life spans are expressed as average life span ± SEM consolidated for all the life span experiments. Full data with the number of experiments (N) and number of animals (n) are reported in Table S1 and S3.

***BDR Lifespan***

BDR was performed as previously described (Chamoli et al., 2014). The OP50 BDR media was prepared as described (Chamoli et al., 2014).Well-fed gravid adult worms were bleached and eggs were then kept on a 90 mm NGM plate seeded with OP50 bacteria. When the worms reached young adult stage, FUDR (100 mg/L) was added to each plate to arrest progeny development. After about 24 hours, worms were transferred to a single well of a 12-well cell-culture plate containing 1 ml (30 worms per well) of S-basal/cholesterol/antibiotics solution with FUDR (100 mg/L). The plate was kept on a 96 well shaker maintained at 20 °C and 300 rpm for 1h to remove adhering bacteria. During this time, appropriately diluted bacterial suspension for the lifespan were added to the 12-well cell culture plates (1 ml solution per well along with FUDR at 100 mg/L). After 1h, 10 worms per well were moved from the S-Basal to the diluted bacterial suspension using a glass pipette connected to a P200 pipetteman. Worms were moved to fresh bacterial solutions after every 3-4 days at which point they were scored for movement by prodding using a platinum wire. Worms that did not respond to gentle prodding with a worm pick were scored as dead and removed; responsive worms were returned to the experiment. During experiments, plates were maintained at 20°C in an incubator shaker at rotation of 100 rpm. Range of O.D. used: 3.0, 1.0, 0.5, 0.25 and 0.125.

***Dauer***

The *daf-2(-), daf-16(-);daf-2(-);daf-16(a),* or *daf-16(-);daf-2(-);daf-16(f)* animals were grown at 15°C and following hypochlorite treatment, eggs were placed on RNAi plates. RNAi of *daf-16* was used as control. The eggs were then incubated at 22˚C for 72 hours after which they were scored for dauers. Dauers were confirmed by treating the animals with 1% SDS for an hour. For the *daf-16(-);daf-2(-);daf-16(f)* strain, the assay was performed at 21°C.

***Reporter Assays***

The promoter-gfp/rfp strains were maintained at 20 ^o^C. Following hypochlorite treatment of gravid adults, eggs were grown on different RNAi bacteria at 20°C till they reached L4-YA. The worms were photographed using Axioimager M2 (Zeiss, Germany) and fluorescence intensity quantified using NIH ImageJ software. Fluorescence intensity is represented as percentage fluorescence of reporter strain in wild-type or *daf-2(-)* background grown on control RNAi. For duration control experiments, the *daf-2(-);Psod-3::gfp* worms were maintained, and grown at 15 ^o^C till L2 and then shifted to 25 ^o^C for 18 h. Subsequently, they were shifted back to 15 ^o^C for 12 h before being photographed under fluorescence microscope. Fluorescence intensity is represented in arbitrary units. The experiment was done twice with n >25.

***Generation of transgenic animals***

Transgenic worms were generated using a Microinjection setup consisting of Nikon TiS inverted microscope fitted with Eppendorf Femtojet Express and Transferman NK2.

***zfp-1(2a)* promoter**

The 1kb promoter region upstream of start codon of *F54F2.2a* was amplified using a forward primer (5' acagtcgacGAT TGTGATGGTGGTTTGGTGA 3') and reverse primer (5' cctctagaTTTTCAGCAATTTCGGGGGACT 3') using Hifidelity PCR system (Kapa Biosystems, USA) and cloned into pPD95.75 using *SalI* and *XbaI* restriction sites. The recombinant plasmid was linearized using S*alI,* purified using PCR purification kit (Qiagen, USA) and injected at a concentration of 10ng/µl into the syncytial gonad of wild-type worms along with 75 ng/µl pRF4 (*rol-6)* co-injection marker. Transformants were selected based on rolling phenotype, and GFP expression was observed under a fluorescence microscope. Two independent lines carrying extra chromosomal arrays were obtained and all gave similar patterns of GFP expression.

***zfp-1(2c)* promoter**

The 0.5 kb promoter region upstream of start codon of *F54F2.2c* was amplified using a forward primer (5' cgcaagcttGTTGTTTCGTCTGCGCTTCTT 3') and reverse primer (5' acacctgcaggATGCAACCTGGGTGCCGG 3') using Hifidelity PCR system (Kapa Biosystems, USA) and cloned into pPD95.75 using *HindIII* and *PstI* restriction sites. The recombinant plasmid was linearized using *HindIII*, purified using PCR purification kit (Qiagen, USA) and injected at a concentration of 24 ng/µl into the syncytial gonad of wild-type worms along with 75 ng/µl pRF4 (*rol-6)* co-injection marker.

***gfl-1* promoter**

The 0.5 kb promoter region upstream of start codon of M04B2.3 (*gfl-1)* was amplified using a forward primer (5' cgcaagcttGGTGTCTACGTTTAAACGCA 3') and reverse primer(5' gcGGATCCGACTAGTTGTGCTGAAAAATAAC 3') using Hifidelity PCR system (Kapa Biosystems, USA) and cloned into pPD95.75 using *HindIII* and *BamHI* restriction sites. The recombinant plasmid was linearized using *HindIII*, purified using PCR purification kit (Qiagen, USA) and injected at a concentration of 20 ng/µl into the syncytial gonad of wild-type worms along with 75 ng/µl pRF4 (*rol-6)* co-injection marker.

***RNA isolation and quantitative real-time PCR***

Synchronized worms were grown on different feeds (OP50 or RNAi) till L4-YA stage (unless otherwise mentioned) after which they were collected in M9 buffer and frozen in Trizol (Invitrogen, USA). The worms were lysed by vigorous vortexing. RNA was purified by phenol:chloroform:isoamylalcohol extraction followed by ethanol precipitation. The concentration and the purity of the RNA were determined using NanoDrop 2000 (Thermo Scientific, USA). Alternatively, the quality of the ribosomal 28 S and 18 S on an agarose gel was used as a measure of integrity and the absorbance at 260/280 nm was used to determine quantity. Gene expression levels were determined by quantitative real time PCR (QRT-PCR) using the DyNAmo Flash SYBR Green mastermix (Thermo Scientific, USA) and Realplex PCR system (Eppendorf, USA) according to manufacturer’s specifications. Relative gene expression was determined after normalizing data to actin. Statistical analysis was performed using SigmaPlot 10.0 (Systat software). All the primers used are listed in Table S2.

***ChIP-PCR***

ChIP was performed as described previously (Kumar et al., 2015; Oh et al., 2006). Briefly, worms were grown as a mixed culture on ~25 petriplates (150 mm) and harvested. Around 2 ml of compact worm slurry was lysed using a glass homogenizer in cross-linking buffer (1% formaldehyde in 1X PBS), followed by incubation for 20 min at RT after which the reaction was quenched with 200 μl of freshly prepared 2.5 M glycine. Following centrifugation, the worm pellet was washed thrice with 1X PBS containing the proteinase inhibitor cocktail (Sigma, USA). The pellet was frozen in liquid nitrogen and later resuspended in 2 ml of SDS lysis buffer (1% SDS, 10 mM EDTA and 50 mM Tris, pH 8.1 containing proteinase inhibitor cocktail) and slurry sonicated using Diagenode sonicator (maximum setting, 25 cycles). After centrifugation, the supernatant was diluted four times using ChIP dilution buffer (0.01% SDS, 1.1% Triton X-100, 1.2 mM EDTA, 16.7 mM Tris-HCl, pH 8.1 and 167 mM NaCl). Lysate equivalent to 2 mg protein was precleared with either 50 μl of salmon sperm DNA/protein A agarose beads (Milipore, USA) or 25 μl of Dynabeads protein A/protein G mix (Life Technologies, USA) for 1 h at 4 ^o^C and then incubated with 25 μl of anti-DAF-16 (raised against the soluble N-terminal half of the protein)(Kumar et al., 2015) or 5µg anti-GFP (mAb 3E6, Invitrogen, USA) for 12–16 h at 4 ^o^C, followed by incubation with 50 μl of the agarose beads for 2 h ) or 25 μl of Dynabeads protein A/protein G mix at 4 ^o^C. The pelleted or magnetically separated beads were washed with 1 ml of the following solutions: once with LSWB (0.1% SDS, 1% Triton X-100, 2 mM EDTA, 20 mM Tris-HCl, pH 8.1, 150 mM NaCl), once with HSWB (0.1% SDS, 1% Triton X-100, 2 mM EDTA, 20 mM Tris-HCl, pH 8.1, 500 mM NaCl), once with LCWB (250 mM LiCl, 1% sodium deoxycholate, 1 mM EDTA, 10 mM Tris-HCl, pH 8.1) and three times with 1X TE. The DNA-protein complex was eluted twice with 250 μl of EB (1% SDS, 0.1 mM NaHCO_3_) and pooled. To this, 50 μl of 5 M NaCl was added and the complexes were reverse crosslinked at 65 ^o^C for 12 h. To the mixture, 10 μl of 500 mM EDTA, 20 μl of 1 M Tris-HCl (pH 6.5) and 20 μl of proteinase K (10 mg/ml) was added, and the mixture was incubated at 45 ^o^C for 2 h. The DNA was then purified using phenol-chloroform and resuspended in 10 μl of 1X TE. Following ChIP, qPCR was performed using promoter-specific primers for DAF-16 and PHA-4 target genes.

***ChIP-seq Data Analysis***

ChIP sequencing for DAF-16 was performed as reported (Kumar et al., 2015). DAF-16 Chip-seq-GSE63865 data is available at

<http://www.ncbi.nlm.nih.gov/geo/query/acc.cgi?token=ktsdemoqjjuxpqb&acc=GSE63865>. PHA-4 ChIP-seq data for L3 stage (SRA-NCBI GSE50301) and ZFP-1 ChIP-seq data (seq-JL00006_ZFP1_N2_L3) were downloaded from modENCODE (http://www.modencode.org/) in .sra format. Downloaded data was converted into fastq format using NCBI-recommended SRA toolkit (version 2.2.2a). Converted fastq of replicates were merged and used for further analysis. Reads were aligned to the *C. elegans* genome (WS230) using Bowtie (v0.12.7) (Langmead et al., 2009) with the following parameters: -q -m 1 --best --strata. Mapped reads were used for peak calling and calculation of read density. Enriched peaks were identified using the peak calling algorithm MACS (v1.4.2) (Zhang et al., 2008) using following parameters: --mfold=5,30 --bw=175 -w. Statistically significant peaks (P < 1 X 10 ^-5^) were used for further analysis. To find target genes, PeakAnalyzer (v1.4) (Salmon-Divon et al., 2010) program was used and all genes having peaks within 2 kb of the promoter region were considered for further analysis.

In order to calculate genome-wide read densities, reads were first extended to mean fragment size (194 bp for ZFP-1, 123 bp for PHA-4 and 158 bp for DAF-16) in the 3' direction of the reads to more precisely reveal the true binding position. Next, the number of reads within 25-bp non-overlapping bins was determined across the whole genome and each read count was normalized by the number of uniquely mapped reads in that sample. This procedure was performed for both the immunoprecipitated as well as the input ChIP samples. The input read count was then subtracted from the immunoprecipitated sample read counts for every bin. To compare ZFP-1 and DAF-16 binding at DAF-16 peaks or ZFP-1 and PHA-4 binding at PHA-4 peaks, the normalized genome-wide read counts for the two samples were first quantile-normalized using the preprocessCore R package. The bin overlapping each DAF-16 peak summit and each PHA-4 peak summit (MACS) was identified and the mean normalized read count was calculated for each sample. This process was repeated for 40 bins upstream and 40 bins downstream of the peak summit (±1,000 bp) and the graph was plotted in Microsoft Excel. To better understand the spread of binding, normalized mean data for all four samples was also plotted as heatmap using MeV (v4.9). UCSC genome browser (Kent et al., 2002) was used for visualizing aligned data as wig files.

**References:**

**Chamoli, M., Singh, A., Malik, Y. and Mukhopadhyay, A.** (2014). A novel kinase regulates dietary restriction-mediated longevity in Caenorhabditis elegans. *Aging Cell* **DOI: 10.1111/acel.12218**.

**Hosono, R., Mitsui, Y., Sato, Y., Aizawa, S. and Miwa, J.** (1982). Life span of the wild and mutant nematode Caenorhabditis elegans. Effects of sex, sterilization, and temperature. *Exp Gerontol* **17**, 163-172.

**Kent, W. J., Sugnet, C. W., Furey, T. S., Roskin, K. M., Pringle, T. H., Zahler, A. M. and Haussler, D.** (2002). The human genome browser at UCSC. *Genome research* **12**, 996-1006.

**Kumar, N., Jain, V., Singh, A., Jagtap, U., Verma, S. and Mukhopadhyay, A.** (2015). Genome-wide endogenous DAF-16/FOXO recruitment dynamics during lowered insulin signalling in C. elegans. *Oncotarget*.

**Langmead, B., Trapnell, C., Pop, M. and Salzberg, S. L.** (2009). Ultrafast and memory-efficient alignment of short DNA sequences to the human genome. *Genome Biol* **10**, R25.

**Oh, S. W., Mukhopadhyay, A., Dixit, B. L., Raha, T., Green, M. R. and Tissenbaum, H. A.** (2006). Identification of direct DAF-16 targets controlling longevity, metabolism and diapause by chromatin immunoprecipitation. *Nat Genet* **38**, 251-257.

**Salmon-Divon, M., Dvinge, H., Tammoja, K. and Bertone, P.** (2010). PeakAnalyzer: genome-wide annotation of chromatin binding and modification loci. *BMC Bioinformatics* **11**, 415.

**Stiernagle, T.** (2006). Maintenance of C. elegans. *WormBook*, 1-11.

**Yang, J. S., Nam, H. J., Seo, M., Han, S. K., Choi, Y., Nam, H. G., Lee, S. J. and Kim, S.** (2011). OASIS: online application for the survival analysis of lifespan assays performed in aging research. *PLoS One* **6**, e23525.

**Zhang, Y., Liu, T., Meyer, C. A., Eeckhoute, J., Johnson, D. S., Bernstein, B. E., Nusbaum, C., Myers, R. M., Brown, M., Li, W., et al.** (2008). Model-based analysis of ChIP-Seq (MACS). *Genome Biol* **9**, R137.
